# Digital Health Monitoring and Intervention Suite for Stress in Frontline Nurses: Prospective Cohort Trial

**DOI:** 10.2196/77818

**Published:** 2026-06-11

**Authors:** Alice Rueda, Josh Martin, Karisa Parkington, Argyrios Perivolaris, Bazen Gashaw Teferra, Gyu Hee Lee, Vanessa K Tassone, Qiaowei Lin, Martin Ivanov, Benjamin Darnell, Lindsay Beavers, Douglas M Campbell, Andrei Torres, Wendy Lou, Anthony Nazarov, Andrea Ashbaugh, Bill Kapralos, Brett Litz, Rakesh Jetly, Adam Dubrowski, Gillian Strudwick, Sridhar Krishnan, Venkat Bhat

**Affiliations:** 1 Intervention Psychiatry Program, St. Michael's Hospital, Unity Health Toronto, Toronto, Ontario, Canada; 2 Department of Electrical, Computer, and Biomedical Engineering, Toronto Metropolitan University, Toronto, Ontario, Canada; 3 Department of Psychiatry, Chobanian and Avedisian School of Medicine, Boston University, Boston, MA, United States; 4 Massachusetts Veterans Epidemiology Research and Information Center, VA Boston Healthcare System, Boston, MA, United States; 5 Allan Waters Family Simulation Program, Unity Health Toronto, Toronto, Ontario, Canada; 6 Department of Physical Therapy, University of Toronto, Toronto, Ontario, Canada; 7 Neonatal Intensive Care Unit, Unity Health Toronto, Toronto, Ontario, Canada; 8 Li Ka Shing Knowledge Institute, St. Michael's Hospital, Unity Health Toronto, Toronto, Ontario, Canada; 9 Department of Pediatrics, Faculty of Medicine, University of Toronto, Toronto, Ontario, Canada; 10 maxSIMhealth, University of Ontario Institute of Technology, Oshawa, Ontario, Canada; 11 Dalla Lana School of Public Health, University of Toronto, Toronto, Canada; 12 Department of Psychiatry, Western University, London, Ontario, Canada; 13 MacDonald Franklin OSI Research Centre, Lawson Research Institute, London, Ontario, Canada; 14 School of Psychology, University of Ottawa, Ottawa, Ontario, Canada; 15 Institute of Mental Health Research, University of Ottawa, Ottawa, Canada; 16 Centre for Addiction and Mental Health, Toronto, Ontario, Canada; 17 Institute of Health Policy, Management and Evaluation, University of Toronto, Toronto, Ontario, Canada; 18 Arthur Labatt Family School of Nursing, Western University, London, Ontario, Canada; 19 Department of Psychiatry, Faculty of Medicine, University of Toronto, 172 St. George Street, Toronto, Ontario, M5R 0A3, Canada, 1 4163604000 ext 76404

**Keywords:** digital health monitoring, digital mental health intervention, stress, virtual reality, psychoeducation, ecological momentary assessments, wearable devices, health care workers, nurses, prospective cohort trial, EMAs

## Abstract

**Background:**

Stress among health care workers (HCWs) contributes to burnout, workforce attrition, and adverse patient outcomes. Although virtual reality (VR), psychoeducation, ecological momentary assessments (EMAs), and wearables have independently shown promise in stress research, no integrated digital suite has combined controlled stress induction, intervention delivery, and longitudinal real-world monitoring in HCWs.

**Objective:**

This study aimed to evaluate the feasibility, engagement, and preliminary effectiveness of a multimodal Digital Health Monitoring and Intervention suite for Stress framework integrating VR simulation, psychoeducation, EMAs, and wearable biometrics. We examined (1) the impact of VR simulation and psychoeducation on stress outcomes and (2) associations between physiological and self-reported mental health outcomes.

**Methods:**

Ninety-nine nurses (mean age 33.7, SD 8.9 yr, 87% female) were enrolled in 2023. We conducted a single-arm prospective cohort study (NCT05923398). Using convenience sampling, participants were recruited from social media advertisements, flyers, and email notices distributed through professional listservs. Participants completed ≥2-week baseline monitoring, a single VR session (2 runs separated by a brief psychoeducation intervention), and 12-week follow-up. In-VR stress was assessed using the Subjective Units of Distress Scale (SUDS) and 4-item Moral Injury Outcome Scale (MIOS-4), with synchronous heart rate variability. Longitudinal outcomes included weekly and biweekly EMAs alongside 70 wearable-derived features. Paired *t* tests, aligned rank transform ANOVA, and Pearson correlations informed study objectives, with P values adjusted for multiple comparisons. Qualitative content analysis classified emotional responses during and after VR.

**Results:**

VR significantly increased subjective stress across checkpoints in both runs, with attenuation in Run B relative to Run A (all *P*<.001). No significant heart rate variability differences were observed between runs (*P=*.15). During VR, 92% (91/99) of participants felt stressed, 36% (36/99) reported anxiety or nervousness, and 51% (50/99)‐78% (77/99) endorsed anger, guilt, shame, and/or betrayal. Most (59/99, 60%) HCWs returned to an emotional baseline post-VR, although 12% (12/99) reported lingering distress. Immediate reliable improvements in anger, guilt, shame, and/or betrayal occurred for 50% (50/99)‐75% (74/99) of participants post intervention. Anxiety (mean −0.53, SD 2.34; *P=*.03) and stress (mean −3.05, SD 11.35; *P=*.01) decreased 2 weeks post intervention, but were not sustained at 12 weeks. Increased sleep restlessness was the only wearable feature showing significant changes (mean 2.46%, SD 5.43; *P_adj_*<.001). In-VR stress correlated with 12-week real-world stress (SUDS: *r*=0.57‐0.58; MIOS-4: *r*=0.58‐0.61; all *P<*.01). Data completion exceeded 90%, with 71% achieving full compliance.

**Conclusions:**

This study moves beyond single-tool interventions to demonstrate the feasibility and preliminary effectiveness of an integrated, multimodal stress platform within a single coordinated framework. This trial demonstrates high engagement, short-term symptom responsiveness, ecological validity, and emotional safety. The framework provides a scalable model for proactive stress identification, skills training, and implementation in high-risk occupational settings. Randomized controlled trials are needed to establish sustained efficacy and optimize deployment for real-world implementation.

## Introduction

### Problem

Stress is a biopsychosocial response elicited by psychological pressures and environmental triggers, commonly observed as a cognitive, emotional, and biological reaction to stressors [1]. Elevated and prolonged levels of stress that exceed an individual’s ability to cope contribute to poor quality of life and adverse health outcomes [2]. More than 20% of adults report experiencing high levels of stress [3], contributing to major economic costs in health care and the workplace (ie, absenteeism and turnover) [4].

The high occupational stress of health care workers (HCWs) [5] was intensified during the COVID-19 pandemic when resources were limited and HCWs were forced to make life-or-death decisions regarding patient care provision [6]. As a result, HCWs experienced heightened stress, anxiety, depression, and burnout compared to prepandemic circumstances [3,6] and are now more likely to leave their profession due to stressful work environments [7]—a major concern given the projected global shortage of 15 million HCWs by 2030 [8]. Addressing HCW stress is an urgent research topic as burnout and stress have broader societal and health care implications, including workforce shortages and negative impacts on patient care. Given the severe impact of stress on the general population and HCWs, there is a pressing need for interventions [9] that reduce stress while effectively monitoring subjective (self-report) and objective (physiological) stress indices for timely intervention.

### Review of Recent Scholarship

Research on stress-reduction interventions demonstrates high tolerability, acceptance, and efficacy among the HCW [10]. Recent meta-analysis indicates two effective approaches in reducing stress: (1) those that aim to modify the experience of stress and (2) those that aim to shift attention away from stress. Psychoeducation is the technique of presenting information on mental health disorders and respective symptoms in a coherent manner. The use of psychoeducation can provide valuable information to individuals on how to cope with their condition. Therefore, psychoeducational interventions hold relevant potential in aiding individuals with their stress reduction and coping mechanisms [11]. Evidence-based interventions focused on the stress experience commonly integrate mindfulness-based stress reduction techniques (eg, self-compassion [12]), unburdening of stressful experiences with a trusted person [13]. Conversely, interventions that direct attention away from stress include various forms of self-care (eg, physical activity [14]) and grounding exercises (eg, diaphragmatic breathing [15]).

Subjectively, acute stress is commonly assessed using momentary ratings such as the Subjective Units of Distress Scale (SUDS) [16] and validated questionnaires indexing perceived stress and anxiety [17]. Physiologically, stress manifests through the autonomic nervous system activation, including reductions in vagal (parasympathetic) tone and increases in sympathetic arousal. Heart rate variability (HRV), particularly root-mean-square of successive differences (RMSSD), is widely used as a noninvasive marker of parasympathetic function and has been associated with stress reactivity, resilience, and occupational burnout [18]. Additional markers such as resting heart rate, sleep efficiency, and recovery indices derived from wearables also reflect the downstream effects of autonomic nervous system dysregulation and allostatic load [19,20].

Recent advancements in technology and digital interventions [21] have revealed the benefits of leveraging virtual reality (VR) [22], video-based psychoeducational interventions [11], ecological momentary assessments (EMAs) [23], and wearable devices [24] for mental health interventions and symptom monitoring. VR-based interventions have been used in mental health, particularly for conditions such as anxiety disorders, posttraumatic stress disorder, and burnout-related distress [25,26]. For instance, VR provides controlled simulation environments that elicit emotions and behaviors comparable to real-world scenarios. VR can also be tailored to address specific research questions [22,27] and serve as a training tool to provide a safe, controlled environment. VR is particularly effective for HCW clinical training and stress reduction [28], especially when combined with psychoeducational intervention videos promoting evidence-based stress reduction techniques (eg, diaphragmatic breathing [15], unburdening [13], and self-compassion [29,30]). While VR is effective for monitoring and reducing stress in controlled environments, other modalities are needed for longitudinal data capture in real-life settings. EMAs are particularly effective for repeatedly sampling participants’ mental well-being in real life, allowing for ecologically valid assessments of well-being. Similarly, accessory-based wearable devices permit nonintrusive, passive monitoring of physiological biometrics and digital biomarkers relevant to mental health (eg, sleep, activity, and heart rate).

Despite the promise of each of these digital tools in isolation, no established digital suites currently integrate digital stressors, therapeutic interventions, and health monitoring systems for a holistic understanding of stress. This mixed methods study addresses this gap by piloting a Digital Health Monitoring and Intervention suite for Stress (DHMI-S; VR technology, psychoeducational intervention, EMAs, and wearable devices) in a single-arm prospective cohort trial. Furthermore, persistent challenges with adherence and compliance in mental health studies remain barriers. In a study of more than 1000 participants, more than half of the participants stopped completing mental health assessments after the first 4 weeks [31]. A separate study of more than 100,000 participants found that median retention was just 5.5 days [32]. As such, this trial implemented engagement strategies (ie, frequent reminders, financial incentives, and engagement specialists) to improve compliance and adherence, addressing poor data completion rates in our pilot trial [33] and low engagement rates commonly reported in mental health interventions and recurrent survey administration.

In this study, we report on the implementation of our novel DHMI-S, combining VR simulations and psychoeducational videos, wearable devices, and EMAs into a single complex intervention. The evaluation of complex interventions requires study designs that balance rigor with real-world feasibility. While randomized controlled trials (RCTs) remain the gold standard for causal inference, they are not always the most practical or ethical choice in early-phase research, particularly for interventions requiring longitudinal digital health monitoring and behavioral engagement [34,35]. Given that this study aimed to assess the feasibility, engagement, and preliminary effectiveness of a multimodal digital health intervention for frontline HCWs, a single-arm prospective cohort design was chosen as the most appropriate first step. This decision aligns with Medical Research Council guidance on evaluating complex interventions, which emphasizes that early-phase feasibility studies should precede RCTs to refine intervention components and optimize adherence strategies [35]. Additionally, randomizing stressed HCWs into a no-intervention control group could raise ethical concerns, potentially leading to higher dropout rates and limiting generalizability. Prospective cohort studies, such as the one used here, allow for real-world evaluation while still capturing meaningful pre-post intervention changes, making them a critical step before investing in an RCT [36].

The psychoeducational intervention in our novel DHMI-S presented three brief, evidence-informed stress-management strategies: (1) diaphragmatic breathing, which is a breathing technique shown to increase parasympathetic tone and reduce anxiety and physiological arousal [37]; (2) unburdening, which aims to reduce moral residue and cognitive load by externalizing distress [38]; and (3) self-compassion interventions that are able to promote self-calming and adaptive emotion regulation while reducing anxiety and burnout symptoms in health professionals [30]. Diaphragmatic breathing targets physiological arousal via vagal activation (observable in RMSSD and resting heart rate), unburdening targets cognitive processing and moral residue by enabling structured disclosure and meaning-making, and self-compassion targets affect regulation and threat appraisal. Together, these techniques aim to engage physiological regulation, cognitive reframing, and emotion regulation pathways, an approach compatible with transdiagnostic coping and psychophysiological models of stress. These techniques have been demonstrated to be good candidates for a VR context and for targeting stress pathways [39].

Given the mixed and emerging evidence for VR-based stress interventions, establishing feasibility is an essential first step before evaluating efficacy. While some wearable metrics from the Oura ring (eg, sleep stages, total sleep time, and interbeat intervals) have demonstrated acceptable agreement with research-grade devices in validation studies, many features are less well validated [40-43]. We therefore treat Oura features as exploratory digital biomarkers and interpret findings cautiously, consistent with recent reviews on consumer wearable validity.

### Hypothesis, Aims, and Objectives

This study addresses the first 2 primary aims of our protocol, which are to evaluate (1) the impact of a VR simulation and psychoeducational intervention on stress outcomes; and (2) the relationship between data collected using the DHMI-S and mental health outcomes. In this study, we hypothesized that (1) our hospital ward VR simulation would elevate HCWs’ self-reported and physiological markers of stress relative to baseline (ie, the beginning of each simulation run); indices of stress would decrease following the psychoeducational intervention video in the second run of the VR session; and indices of stress would decrease in the 2 weeks following the VR session; (2) the stress experienced by participants in the VR simulation would correlate with real-life stress outcomes; changes in mental health outcomes would correlate with changes in wearable features; and high adherence levels would be maintained throughout the full study period (up to 12 weeks post intervention). Future publications will explore the third aim in our protocol to develop exploratory personalized models that predict stress based on passively collected data.

## Methods

### Ethical Considerations

This single-arm, prospective cohort trial was registered with ClinicalTrials.gov (NCT05923398) on December 20, 2022, and received Unity Health Toronto Research Ethics Board approval (22‐279) in April 2023. All participants provided written informed consent before participation and were protected through a deidentified process. Participants’ identification has been replaced with an identifier in the format of “DND2-1234567” and assigned a study email address with dnd2-1234567@ippregistry.ca. The identifier was prefixed with the project code, followed by 7 digits generated by a random number generator. No identification of individual participants, including images, was contained in the paper or supplementary material. Each participant was rewarded up to a total of CAD $370 (US $285) and the wearable device (Oura Ring) if they fulfilled the data adherence requirements.

### Study Design

**Figure 1.**
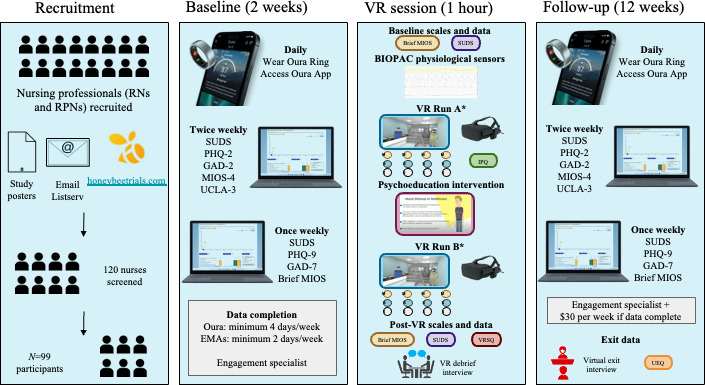
Study design including recruitment, pre–virtual reality session, 1-hour virtual reality session, and 12-week follow-up. Brief MIOS: Brief Moral Injury Outcome Scale; EMA: ecological momentary assessment; GAD-2: 2-item Generalized Anxiety Disorder; GAD-7: 7-item Generalized Anxiety Disorder; IPQ: Igroup Presence Questionnaire; MIOS-4: 4-item Moral Injury Outcomes Scale; PHQ-2: 2-item Patient Health Questionnaire; PHQ-9: 9-item Patient Health Questionnaire; SUDS: Subjective Units of Distress Scale; UCLA-3: UCLA 3-item Loneliness Scale; UEQ: User Experience Questionnaire; VR: virtual reality; VRSQ: Virtual Reality Sickness Questionnaire.

Figure 1 outlines the study design. EMA and wearable data collection started upon enrollment and continued for 14 weeks. HCWs were recruited through social media advertisements (Honeybee Hub, Inc), flyers posted at St. Michael’s Hospital, and email lists. Candidates were screened according to the inclusion and exclusion criteria, and enrolled on a first-come, first-served basis. Upon enrollment, participants were fitted with the wearable for passive data collection and administered 4 weekly and 4 twice-weekly EMAs. The VR simulation and psychoeducational intervention were delivered in a single session 2 weeks or more after enrollment. Participants underwent 2 runs (A and B) within-VR simulated stress with a psychoeducational video in between. Physiological signals and EMAs (SUDS and MIOS-4) were recorded during the whole VR session, followed by a semistructured interview. The Igroup Presence Questionnairewas administered after Run A, and Virtual Reality Sickness Questionnaire was administered after Run B . After 12 weeks of continuous monitoring using wearable devices and EMA reporting under a comprehensive engagement strategy, the participants completed the program with a semistructured exit interview.

### Inclusion and Exclusion Criteria

Inclusion criteria required all participants to be a registered practical nurse or a registered nurse currently employed at an Ontario health care institution and to own a smartphone. Exclusion criteria included HCWs with elevated anxiety or depression (indexed by 7-item Generalized Anxiety Disorder [GAD-7] scores ≥15 and/or 9-item Patient Health Questionnaire [PHQ-9] scores ≥20), a history of seizures (except febrile seizure), and/or current use of an electronic medical device.

### Participant Characteristics

HCWs were predominantly female, capturing a representative distribution across age (22‐66, mean 33.7, SD 8.9 y) and nursing experience (Table 1). Of the 86 females, 83 identified as women, 1 as nonbinary, and 2 preferred not to specify. Most participants were White or Asian (86/99, 86%) and from the Greater Toronto Area (88/99, 87%). Consistent with our eligibility criteria, participants presented with minimal-to-mild anxiety and depression symptoms at screening. Table 1 provides the mental health screening (GAD-7 and PHQ-9) of all participants who completed the trial protocols.

**Table 1.** Participant demographics and mental health symptoms prior to the study^a^.

Category	Participants (N=99)
Sex, n (%)	
Female^b^	86 (86.8)
Male	13 (13.1)
Race, n (%)	
Asian	40 (40.4)
Black	5 (5.1)
White	46 (46.5)
Other	8 (8.1)
Years of nursing experience, n (%)	
<5 years	35 (35.4)
5‐10 years	33 (33.3)
>10 years	30 (30.3)
Not reported	1 (1)
Place of residence, n (%)	
GTA^c^	88 (88.9)
Ontario, non-GTA	9 (9.1)
Not reported	2 (2)
Baseline mental health symptoms, mean (SD)	
Anxiety: GAD-7^d^, out of 14	4.703 (3.560)
Depression: PHQ-9^e^, out of 19	5.762 (4.255)

a Data are in n (%) or mean (SD).

b Of the 86 females, 83 identified as women, 1 identified as nonbinary, and 2 preferred not to specify.

c GTA: Greater Toronto Area.

d Exclusion cutoff for GAD-7 ≥15.

e Exclusion cutoff for PHQ-9 ≥20.

### Sampling Procedures

Registered nurses and registered practical nurses were recruited through social media advertisements, flyers posted at St. Michael’s Hospital, and email notices distributed through professional listservs relevant to Ontario-based nurses. The trial was conducted at St. Michael’s Hospital between May and December 2023. Overall, 437 HCWs expressed interest in this study, of whom 119 were screened. Of these, 101 participants were enrolled using a convenience sampling recruitment procedure: one participant withdrew while the screening window was still open and was replaced, while another participant withdrew after the screening window closed and was not replaced. Thus, 99 participants completed the VR simulation. Two participants withdrew before the exit interview, which left 97 participants who completed the study in its entirety. However, the results from the exit interview are not presented herein, so a final sample of 99 participants (Table 1) is presented in this study unless otherwise specified.

### Sample Size, Power, and Precision

The target sample size for this study was 100 HCWs, with sample size estimates determined based on post hoc analyses of our pilot study [44]. For more details, see the study by Martin et al [45].

### Measures and Covariates

Primary outcomes included self-reported and physiological indices of stress measured within the VR simulation, as well as longitudinal real-world stress outcomes. In-VR subjective stress was assessed using the SUDS and the 4-item Moral Injury Outcome Scale (MIOS-4)—a self-report measure assessing acute moral distress, including feelings of shame and trust violation. Participants were presented with Likert rating scales within the VR environment at 4 predefined checkpoints (Table 2) during each run (Run A and Run B) and asked to indicate their level of distress (SUDS: 0‐100; MIOS-4: 0‐10). In-VR physiological stress was indexed using HRV, specifically the RMSSD, derived from continuous electrocardiography (ECG) recordings collected using BIOPAC MP160 equipment. HRV was calculated using 5-minute windows with 95% overlap to capture dynamic autonomic responses during the simulation. Real-world stress outcomes were assessed through weekly SUDS and Brief Moral Injury Outcome Scale (Brief MIOS) measures, which were averaged across the 2-week preintervention baseline and postintervention periods (2-wk and 12-wk follow-up).

**Table 2.** Summary of checkpoints presented throughout the VR^a^ simulation for runs A and B.

Checkpoint	Description
1A	Begin - baseline; immediately before the participant begins the simulation.
2A	Code blue - immediately before the participant is required to choose which of 2 patients to attend to in a Code Blue situation.
3A	Choice - immediately after the participant chooses which patient to attend to in the Code Blue situation.
4A	Lost patient - immediately after the participant has been informed that the unattended patient has died and the simulation has ended.
1B	Begin - baseline; same as checkpoint 1A (immediately follows the psychoeducational intervention).
2B	Code blue - same as checkpoint 2A.
3B	Choice - same as checkpoint 3A, except the participant has been automatically directed to attend to the same patient as in session A.
4B	Lost patient - same as checkpoint 4A.

a VR: virtual reality.

Secondary mental health outcomes were collected through full-form weekly EMAs (depression: PHQ-9; anxiety: GAD-7; and stress: SUDS) and abbreviated biweekly EMAs (depression: PHQ-2; anxiety: 2-item General Anxiety Disorder [GAD-2]; loneliness: UCLA 3-item Loneliness Scale [UCLA-3]; and stress: SUDS and MIOS-4). Pre-post changes were examined using paired analyses, and Reliable Change Index (RCI) calculations were conducted to determine clinically meaningful improvement or worsening for selected measures.

Passive physiological and behavioral data were collected continuously using the wearable device (Oura Ring Generation 3). A total of 70 device-specified features were extracted across 3 domains: activity (eg, movement and step counts), readiness (eg, resting heart rate and HRV-derived indices), and sleep (eg, total sleep time, sleep efficiency, sleep onset latency, and sleep restlessness). These wearable-derived metrics were treated as exploratory digital biomarkers.

Qualitative data were collected through a semistructured VR debrief interview immediately following the VR session and a separate exit interview conducted at study completion. Audio and video recordings were transcribed for subsequent analysis.

Covariates included demographic variables (age and gender identity, ethnicity, and geographic region), professional characteristics (years of nursing experience), and baseline mental health screening scores (PHQ-9 and GAD-7). Analytical covariates included VR run (Run A vs Run B), baseline checkpoint values within each run, and adherence metrics (EMA completion rates and wearable data synchronization).

### Data Collection

Data were collected using a multimodal approach integrating VR response selection, self-report questionnaires, wearable physiological monitoring, laboratory-grade ECG acquisition, and qualitative interviews. Following written informed consent, participants completed a minimum 2-week baseline monitoring period during which they wore the Oura Ring Generation 3 continuously to passively collect sleep, activity, and readiness metrics, and completed scheduled EMAs via electronic survey links. Weekly full-form EMAs (SUDS, PHQ-9, GAD-7, and Brief MIOS) were administered on Saturdays, and biweekly short-form EMAs (SUDS, PHQ-2, GAD-2, UCLA-3, and MIOS-4) were administered on Mondays and Thursdays. EMA data were captured electronically using Greenspace [46]. A detailed and visual overview of the study design and procedural sequence is shown in Figure 1.

The VR session was conducted following a standard operating procedure for all participants. The simulation and intervention video were presented using a Meta Quest 2 Reality Labs head-mounted VR display and Meta Quest 2 hand controllers to interact with the environment. The VR environment depicted a stressful hospital ward scenario, with 2 critically ill patients whom participants had to care for amid staff shortages and the absence of lifesaving equipment. Participants were forced to choose a patient to save. Each participant completed 2 identical ~10-minute VR runs, before (Run A) and after (Run B) the psychoeducational intervention. The brief (8-min) psychoeducational video for HCWs incorporated information about stress and moral distress alongside 3 evidence-based stress-reduction techniques: diaphragmatic breathing exercises, unburdening, and self-compassion (including self-care). During each VR run, subjective stress (SUDS and MIOS-4) was collected at predefined checkpoints (Table 2) within the virtual environment. Simultaneously, physiological biomarkers were monitored throughout the VR session using BIOPAC MP160 equipment (BIOPAC Systems Inc), sampling at a frequency of 2000 Hz. The BN-RSPEC BioNomadix model run in conjunction with AcqKnowledge 5 software (BIOPAC Systems Inc) was used to acquire raw ECG recordings and allowed to derive heart rate variability (RMSSD) as an index of autonomic stress response.

Immediately following the VR session, participants completed a semistructured debrief interview guided by the Promoting Excellence and Reflective Learning in Simulation (PEARLS) health care debriefing tool [47]. Interviews probed participants’ experiences, feelings, and beliefs regarding the simulation, assessed retention of psychoeducation material, and allowed open-ended feedback. Participants then continued wearable monitoring and EMA completion for 12 weeks post intervention. To enhance adherence and data completeness, automated reminders, engagement specialist follow-up, and biweekly compensation were implemented. Wearable and EMA data were synchronized and downloaded for analysis at prespecified time points.

At the end of the 12-week postintervention period, a semistructured exit interview was conducted over Zoom (Zoom Video Communications), and participants completed user experience surveys for the wearable device and EMAs. Exit interviews explored participants’ experiences since the VR session, recall and perceived usefulness of the stress-management techniques, acceptability of wearable and EMA procedures, and overall suggestions for improvement.

### Conditions and Design

In line with the UK Medical Research Council guidelines for complex interventions, we conducted a single-arm cohort study to evaluate the feasibility and preliminary evidence for the DHMI-S. We followed the STROBE (Strengthening the Reporting of Observational Studies in Epidemiology) checklist for cohort studies (Checklist 1) [48].

A brief overview is provided below; please see the previously published protocol paper [45] for further details and multimedia materials. Participants were instructed to wear the wearable device continuously throughout the study period, including during work shifts and sleep, and to synchronize data regularly using the companion mobile app.

During the second VR run (Run B), participants were encouraged to apply any of the stress-management strategies taught in the preceding psychoeducation module (diaphragmatic breathing, unburdening, or self-compassion). However, the study did not include an objective or time-stamped measure of strategy use within VR. Application of these techniques was assessed only through participants’ subjective reports during the in-VR debrief and the exit interview. As a result, we were unable to determine when or how often specific strategies were used during the scenario.

### Engagement Strategies

Three engagement strategies were combined to maximize digital monitoring adherence and participant engagement. First, scheduled email and text reminders encouraged participants to complete EMAs and upload wearable device data. Second, a study engagement specialist provided technical assistance and initiated follow-up phone calls when participants had 3 or more consecutive days of incomplete data. Third, participants accrued gift card compensation biweekly for their participation (please see the previously published protocol paper [45] for details) and were eligible to keep the wearable device if they adhered to more than 50% of compliance protocols, including wearing the ring and synchronizing their data.

### Masking

Masking was not feasible for this study, given the single-arm prospective cohort design. All participants received the VR simulation and psychoeducational intervention, and there was no comparator group or allocation process to blind participants or study personnel to the condition.

### Data Diagnostics

All data diagnostics were prespecified and conducted prior to hypothesis testing to ensure analytic validity and transparency. Due to the complexity of the study, we have provided a summary of the analysis provided in Table 3.

**Table 3.** Summary of the within participant–based statistical analysis used in the study.

Analysis	Method	FWE^a^ correction	Threshold *P* adjusted	N
MIOS-4^b^ in-VR^c^	ART-ANOVA^d^	Bonferroni	.05	99
SUDS^e^ in-VR	ART-ANOVA	Bonferroni	.05	99
HRV^f^ in-VR	ART-ANOVA	Bonferroni	.05	91^g^
2-week pre-post short-form EMAs^h^	*t* test	—^i^^,^^j^	—	99
2-week pre-post extended EMAs	*t* test	—	—	95^k^
2-week pre-post Oura	*t* test	FDR^l^	.05	99
Oura correlations	Pearson correlation	FDR	.05	99
Relating in-VR and real-life stress indices	Pearson correlation	Bonferroni	.05	95
Qualitative content analysis (emotions)	—	—	—	99

a FWE: family-wise error.

b MIOS-4: 4-item Moral Injury Outcome Scale.

c VR: virtual reality.

d ART-ANOVA: aligned rank transform ANOVA.

e SUDS: Subjective Units of Distress Scale.

f HRV: heart rate variability.

g Eight of the electrocardiography recordings have poor quality and were excluded from the analysis (n=8).

h EMA: ecological momentary assessment.

i Not available.

j Not applicable.

k Four participants have missing responses (n=4).

l FDR: false discovery rate.

Participants were excluded from specific analyses only if they lacked usable data for the relevant outcome. For example, HRV analyses required artifact-free ECG recordings during VR; participants with irreparable ECG signal noise or insufficient R-peak detection were excluded from HRV-specific models but retained for all subjective analyses. No participants were excluded post hoc based on outcome magnitude, intervention response, or statistical influence. Analyses were conducted on all available data consistent with a modified intention-to-observe framework appropriate for single-arm cohort designs.

Missingness was evaluated using Little Missing Completely at Random (MCAR) test [49]. EMA noncompletion was treated as missing at the observation level (all-or-none survey completion), and wearable data gaps were attributed to device nonwear or synchronization failure. Given the longitudinal and exploratory nature of the study, no single-value imputation (eg, mean substitution or last observation carried forward) was performed. Analyses were conducted using available-case data. Pre-post comparisons required both pre- and postintervention values for inclusion in paired tests. Correlation analyses were restricted to participants with complete data for the variables under investigation. Because the primary focus was on feasibility and signal detection rather than population parameter estimation, multiple imputation was not implemented.

**Figure 2.**
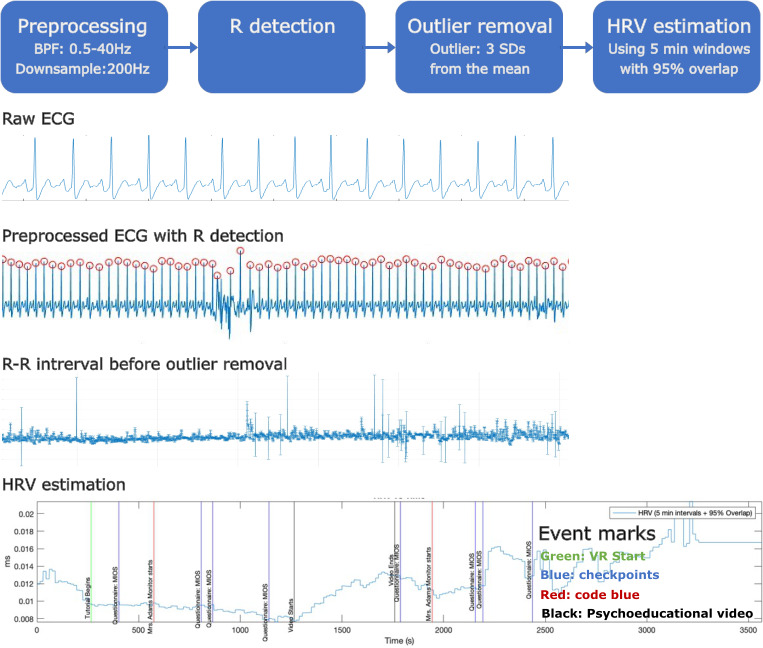
Heart rate variability (root-mean-square of successive differences) feature extraction from the electrocardiography signals. ECG: electrocardiography; HRV: heart rate variability; VR: virtual reality.

For physiological data, preprocessing steps were applied prior to analysis using MATLAB (version 2022b; MathWorks). Quality assurance checks were implemented by visual inspection of ECG signal morphology, then preprocessed (bandpass filtered and downsampled), and the Pan-Tompkins algorithm [50] was used to detect the QRS complex from the ECG signals. HRV was extracted from the preprocessed ECG signal based on R-wave peak locations using a 5-minute 95% overlapping window to calculate the RMSSD of heartbeats for HRV, as illustrated in Figure 2. (A) Four steps are used in extracting the HRV (RMSSD) feature, including preprocessing (bandpassing the signal between 0.05 Hz and 40 Hz and downsampling to 200 Hz), R-wave detection, estimating R-R interval and outlier removal, and calculating HRV using a 5-minute window. (B) Quality-checked raw ECG signals were used in the feature extraction. (C) The location of the R-wave was detected as illustrated by red circles. (D) Outliers due to missing R-waves were identified and removed before HRV estimation. (E) HRV estimation across the whole duration of the VR session using a 5-minute window size with 95% overlapping. If distributional skewness was observed in physiological variables, transformations (eg, logarithmic transformation of RMSSD) were considered to improve normality; however, analyses were ultimately conducted on appropriately scaled values following preprocessing. Implausible R-R intervals and physiologically impossible values were removed during preprocessing.

For wearable-derived features, extreme values were retained unless clearly attributable to device malfunction or recording error. For self-report scales, no observations were excluded as outliers unless values exceeded scale bounds (which did not occur). In-VR SUDS scores were downsampled offline to a truncated 0‐10 Likert range for analysis to align with EMA-based SUDS responses. No other transformations were applied to bounded psychometric scales (eg, PHQ-9, GAD-7, and MIOS-4), as these were analyzed using methods robust to minor deviations from normality. Pre-VR intervention and post-VR intervention values were averaged over the 2 weeks immediately preceding (pre-VR) and immediately following (post-VR) the VR session. Biweekly (twice-weekly) short-form EMA and extended-weekly scores were averaged across weeks, and duplicated wearable metrics were excluded. Sensitivity checks were conducted to confirm that extreme but plausible values did not unduly influence parametric results.

Normality of continuous variables was assessed using the Shapiro-Wilk test and visual inspection (histograms and Q-Q plots). Homogeneity of variance assumptions were evaluated where applicable. For repeated-measures in-VR analyses, nonparametric aligned rank transform ANOVA (ART-ANOVA) was used when distributional assumptions were violated. Paired *t* tests were used for approximately normally distributed pre-post comparisons; nonparametric alternatives were applied where appropriate.

### Analytic Strategy

All statistical analyses were conducted using RStudio (version 4.3.3; Posit). Little MCAR [49] was used to determine the random nature of the missing data. Shapiro-Wilk test was used to test normality. Nonparametric methods were used for data that was not normally distributed. Changes in EMA and wearable data were analyzed using within-participant paired *t* tests alongside measures of effect size (Cohen *d*); within-participant nonparametric repeated-measure analyses were conducted on in-VR MIOS-4, SUDS, and HRV by using ART-ANOVA, applying an aligned rank (AR) transform to the data, preparing it for nonparametric ANOVA against each participant’s baseline (checkpoint 1 for each run). This approach allows for the evaluation of the effects of the intervention in a structured, hypothesis-driven manner while ensuring appropriate handling of repeated measures data. Partial eta-squared (η_p_^2^) was used to understand the overall effect size within and between VR runs. RCI [51,52] was used to determine the number of participants who showed improvement, worsening, or no reliable change following the psychoeducational intervention. Data were assumed to have moderate internal consistency reliability (*r_xx_*=0.8) and thresholded indices to 1.96 SD (α=.05, 2-tailed) for improvement and worsening [51]. To address hypothesis 1a, the 2 runs were analyzed separately for the SUDS, MIOS-4, and HRV. For each run, checkpoint 2 versus 1, checkpoint 3 versus 1, and checkpoint 4 versus 1 were compared using within-participant nonparametric repeated measures ART-ANOVA and an AR pairwise contrast was used as the post hoc analysis. Bonferroni correction was applied to adjust *P* values for the 3 pairwise comparisons within each run. To evaluate hypothesis 1b, the 3 indices of stress (MIOS-4, SUDS, and HRV) were analyzed. First, the differences between checkpoints 2 versus 1, 3 versus 1, and 4 versus 1 were calculated. These differences were then compared across runs using within-participant ART-ANOVA, an AR pairwise contrast for post hoc analysis, with Bonferroni correction applied to adjust *P* values for the 3 pairwise comparisons. At the individual level, RCI was used to compare the checkpoints just before (Run A checkpoint 4) and just after (Run B checkpoint 1) the psychoeducational intervention. For hypothesis 1c, pairwise *t* tests were used to compare 2-week averages pre- and post-VR sessions on all 9 EMA scales with no *P* value adjustment and wearable features with FDR to adjust *P* values. RCI was also used to compare the 2-week average before and after the VR session.

To address hypothesis 2a, we investigated the correlation between EMA and wearable features as well as in-VR scales (SUDS and MIOS-4) with a 12-week average post-VR EMA with Bonferroni *P* value adjustment. Thresholding at *P*=.05, Pearson correlations assessed relationships between changes in EMA and wearable data before and after the VR intervention, and were adjusted with FDR for family-wise error correction. A summary of the statistical analysis is provided in Table 3.

Audio recordings from the semistructured VR debrief interviews were transcribed using Whisper (OpenAI) and manually reviewed for accuracy and simple transcription conventions [53] by 2 researchers. Frequencies of emotions elicited during, and persisting after, the VR simulation were synthesized using qualitative content analysis. Specifically, 2 researchers independently reviewed the transcripts and classified whether target emotions (stress, anger, guilt, shame, and betrayal) were experienced (1), not experienced (0), or insufficiently described to code (NA). Other spontaneously reported feelings (eg, frustration, anxiety, and annoyance) were identified through data-driven inductive category development and recorded in researcher notes. Thereafter, coded responses were compared (Cohen κ=0.63‐0.88), and disagreements were resolved through discussion and consensus.

## Results

### Participant Flow

**Figure 3.**
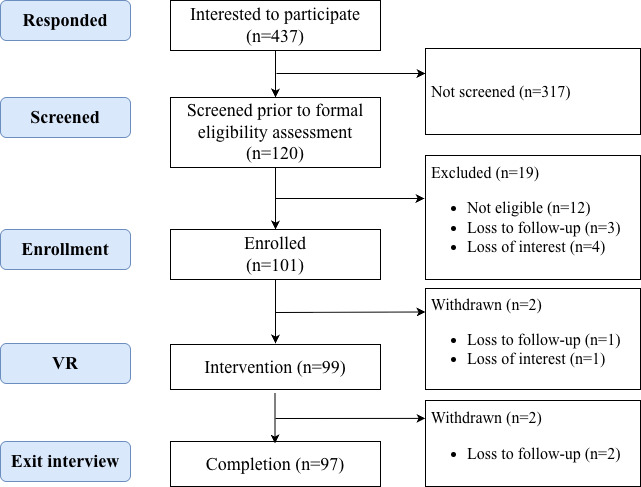
Participant flow diagram.

Overall, 437 HCWs expressed interest in this study, of whom 119 were screened (Figure 3). Of these, 101 participants were enrolled: one participant withdrew while the screening window was still open and was replaced, while another participant withdrew after the screening window closed and was not replaced, which left 99 participants who completed the VR simulation. Two participants withdrew before the exit interview, which left 97 participants who completed the study in its entirety. However, the results from the exit interview are not presented herein, so a final sample of 99 participants is presented in this study unless otherwise specified.

### Statistics and Data Analysis: Self-Reported Stress in VR

**Figure 4.**
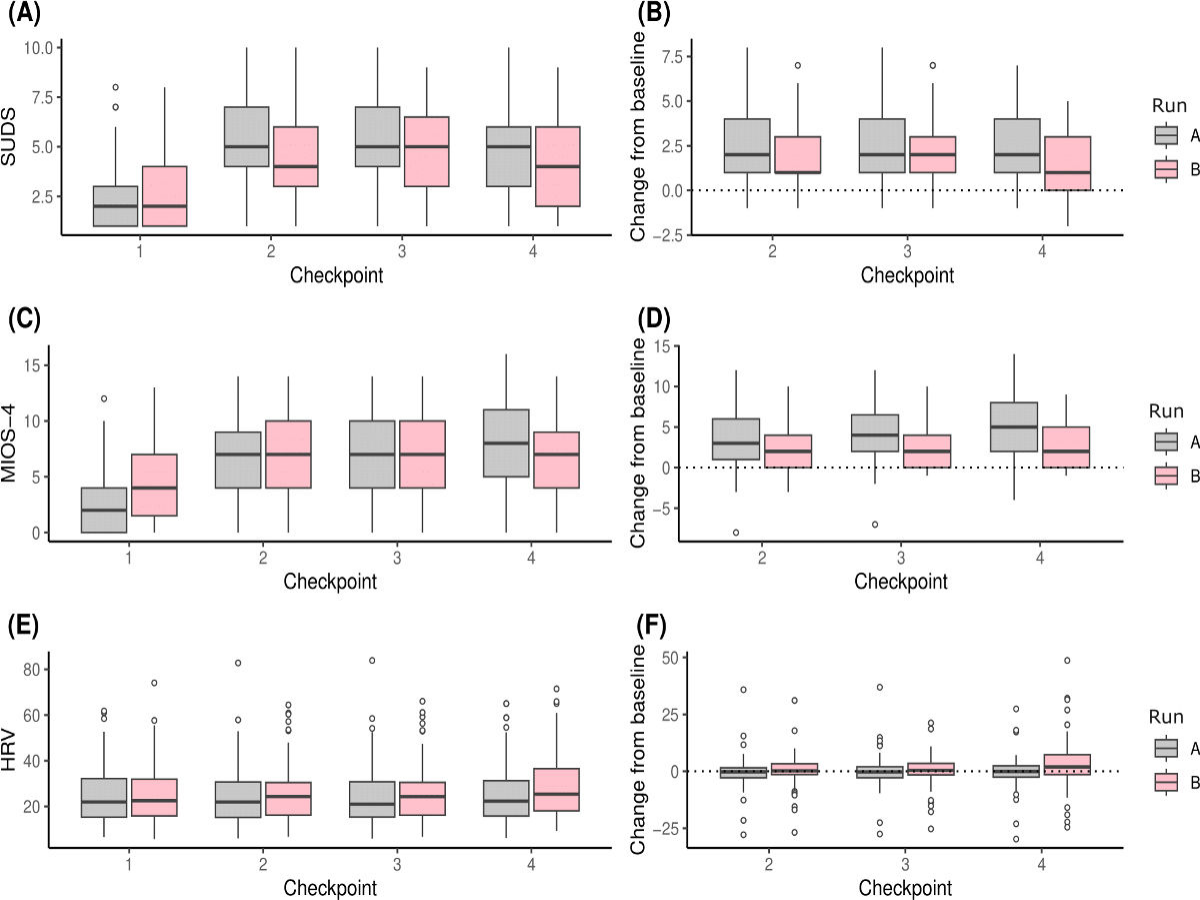
Box plots of stress indices across virtual reality simulation checkpoints (1-4) and runs A and B.

The box plots of Figure 4 illustrate the differences in stress indices across virtual reality simulation checkpoints (1-4) and Runs A and B. At 4 time points for each run, the box plots illustrate the SUDS, MIOS-4, and HRV obtained from participants before (Run A) and after (Run B) the psychoeducational intervention. (A) Subjective Units of Distress Scale scores across checkpoints in Runs A and B (N=99). (B) Changes in SUDS from baseline. (C) MIOS-4 scores across checkpoints (N=99). (D) Changes in MIOS-4 from baseline. (E) Changes in HRV across checkpoints in Run A and B (n=91). (F) Changes in heart rate variability from baseline. Heart rate variability was determined by the root-mean-square successive differences in heartbeat over subsequent 5-minute windows.

Within both runs of the VR simulation, SUDS (Run A: *F*_3,294_=132.11, *P*<.001, $\eta_{p}^{2}$=0.57, 95% CI 0.52‐1.00; Run B: *F*_3,294_=84.95, *P*<.001, $\eta_{p}^{2}$=0.46, 95% CI 0.40‐1.00) and MIOS-4 (Run A: *F*_3,294_=120.27, *P*<.001, $\eta_{p}^{2}$=0.55, 95% CI 0.49‐1.00; Run B: *F*_3,294_=66.69, *P*<.001, $\eta_{p}^{2}$=0.40, 95% CI 0.33‐1.00) scores differed by checkpoint (Figure 4; Table S1 in Multimedia Appendix 1). This was driven by increased SUDS (all *P<.*001) and MIOS-4 (all *P<*.001) scores at checkpoints 2, 3, and 4, relative to checkpoint 1 (ie, baseline), indicating that the VR simulation elicited subjective feelings of stress (Table 4). No significant changes were observed in HRV, a physiological indicator of stress, across checkpoints in Run A (*F*_3,270_=0.63, *P*=.59, $\eta_{p}^{2}$=0.007, 95% CI 0.00‐1.00). A small, but significant, difference was observed between checkpoints in Run B (*F*_3,270_=8.93, *P*<.001, $\eta_{p}^{2}$=0.09, 95% CI 0.04‐1.00), which was driven by a difference in HRV between checkpoints 4B and 1B (*P<.*001).

To determine whether the stress levels elicited by the VR simulation differed between Run A and Run B, we compared the changes from baseline in each run. The changes from baseline differed significantly between Run A and Run B on both the SUDS (*F*_1,490_=17.46, *P*<.001, $\eta_{p}^{2}$=0.03, 95% CI 0.03‐1.00) and the MIOS-4 (*F*_1,490_=32.11, *P*<.001, $\eta_{p}^{2}$=0.06, 95% CI 0.03‐1.00; Figure 4 and Table 4). Post hoc comparisons showed that changes from baseline were lower at all checkpoints in Run B (all *P<.*001 for MIOS-4 and SUDS), indicating that participants’ stress levels increased less on the second run. However, the average HRV did not significantly differ between runs (*F*_1,450_=3.16, *P*=.15, $\eta_{p}^{2}$=0.007, 95% CI 0.00‐1.00).

At the individual level, RCI revealed the immediate effect of the psychoeducational intervention with improvements among the participants. Both MIOS-4 and SUDS have decreased scores with RCI indicating improvement for 37-out-of-99 (37%) and 41-out-of-99 (41%) participants, respectively. Of the 91 participants who passed the quality-checked HRV data, only 2-out-of-91 (2.20%) have increased HRV. No participant has worsened conditions in MIOS-4, SUDS, or HRV. The remaining participants showed no reliable change.

**Table 4.** Comparative analyses of self-report (MIOS-4^a^ and SUDS^b^, N=99) and physiological (HRV^c^, n=91) measures of stress across VR^d^ checkpoints (1-4) and runs (A, B)^e^.

	SUDS (N=99)			MIOS-4 (N=99)			HRV-RMSSD^f^ (n=91)^g^			
Hypothesis tests: ART-ANOVA^h^										
Comparison	ΔMedian (IQR)	*F* test (*df*)	$\eta_{p}^{2}$(95% CI)	ΔMedian (IQR)	*F* test (*df*)	$\eta_{p}^{2}$(95% CI)	ΔMedian (IQR)	*F* test (*df*)	*P* value	$\eta_{p}^{2}$(95% CI)
Run A checkpoints 1‐4	2.000 (1.000 to 4.000)	32.11 (3,294)^i^	0.57 (0.52‐1.00)	4.00 (1.000 to 7.000)	120.27 (3,294)^i^	0.55 (0.49‐1.00)	−0.196 (−2.761 to 1.963)	0.634 (3,270)	.59	0.007 (0.00 to 1.00)
Run B checkpoints 1‐4	1.000 (0.000 to 2.000)	4.952 (3,294)^i^	0.46 (0.40‐1.00)	2.00 (0.000 to 4.000)	66.685 (3,294)^i^	0.40 (0.33‐1.00)	0.000 (−0.265 to 1.329)	8.93 (3,270)	<.001	0.09 (0.04 to 1.00)
Run B vs Run A	−0.667 (−1.667 to 0.000)	17.459 (1,490)^i^	0.03 (0.01‐1.00)	−1.33 (−3.17 to 0.000)	32.109 (1,490)	0.06 (0.03‐1.00)	2.495 (−1.072 to 6.226)	3.160 (1,450)	.15	0.007 (0.00 to 1.00)
Post hoc analysis: aligned ranks pairwise contrast										
Comparison	ΔMedian (IQR)	Estimate	95% CI	ΔMedian (IQR)	Estimate	95% CI	ΔMedian (IQR)	Estimate	*P*_adj_	95% CI
2A vs 1A^j^	2.000 (1.000 to 4.000)	135.980^k^	114.636‐157.323	3.000 (1.000 to 6.000)	100.747^k^	79.999 to 121.496	−0.226 (−2.841 to 1.567)	−4.813	1.00	−14.782 to 5.155
3A vs 1A^j^	2.000 (1.000 to 4.000)	143.040^k^	121.697‐164.384	4.000 (2.000 to 6.50)	118.056^k^	97.307 to 138.804	−0.275 (−2.835 to 2.000)	−3.615	1.00	−13.584 to 6.353
4A vs 1A^j^	2.000 (1.000 to 4.000)	119.384^k^	98.040‐140.727	5.000 (2.000 to 8.000)	141.298^k^	120.549 to 162.047	−0.080 (−2.531 to 2.393)	−1.352	1.00	−11.320 to 8.617
2B vs 1B^l^	1.000 (1.000 to 3.000)	100.515^k^	81.327‐119.703	2.000 (0.000 to 4.000)	71.833^k^	54.895 to 88.772	0.240 (−1.413 to 3.290)	5.187	1.00	−8.949 to 19.323
3B vs 1B^l^	2.000 (1.000 to 3.000)	104.439^k^	85.252‐123.627	2.000 (0.000 to 4.000)	79.298^k^	62.360 to 96.236	0.419 (–1.526 to 3.477)	6.648	1.00	−7.488 to 20.785
4B vs 1B^l^	0.000 (0.000 to 0.000)	73.308^k^	54.120‐92.496	2.000 (0.000 to 5.000)	75.253^k^	58.314 to 92.191	0.000 (0.000 to 0.000)	26.341	<.001	12.204 to 40.477
2B-1B vs 2A-1A^j^	−1.000 (−1.000 to 0.000)	−64.753^k^	−101.590 to −27.918	−1.000 (−3.000 to 1.000)	−62.864^k^	−103.321 to −22.406	1.025 (–2.085 to 4.278)	40.780	.97	−16.476 to 98.037
3B-1B vs 3A-1A^k^	−1.000 (−1.000 to 0.000)	−67.788^k^	−104.622 to −30.954	−1.000 (−3.000 to 0.000)	−83.106^k^	−123.564 to −42.648	1.022 (−2.422 to 4.852)	40.670	.98	−16.586 to 97.927
4B-1B vs 4A-1A^m^	−1.000 (−2.000 to 0.000)	−81.470^k^	−118.304 to −44.635	−2.000 (−4.000 to 0.000)	−126.899^k^	−167.357 to −86.441	3.210 (−1.119 to 8.690)	70.077	.02	12.820 to 127.333
Immediate effect of the psychoeducational intervention: Reliable Change Index										
Comparison	Improved	Worsened	No change	Improved	Worsened	No change	Improved	Worsened		No change
4B vs 1B	41	0	58	37	0	62	2	0		89

a MIOS-4: 4-item Moral Injury Outcome Scale.

b SUDS: Subjective Units of Distress Scale.

c HRV: heart rate variability.

d VR: virtual reality.

e All data are nonnormal with *P*<.05 from Shapiro-Wilk test. Aligned rank transform ANOVA was used as the nonparametric repeated measure. Aligned ranks pairwise contrast was used as the post hoc analysis. The estimate reflects the adjusted mean between the 2 groups. *P*<.05 denotes statistical significance. *P*_adj_ = *P* values were adjusted for multiple tests using Bonferroni method.

f RMSSD: root-mean-square of successive differences.

g Four electrocardiography files were not recorded properly and 4 more were rejected due to the quality of the signal.

h ART-ANOVA: aligned rank transform ANOVA.

i *P* <.001.

j Comparisons within Run A were simultaneously tested.

k *P*_adj_<.001.

l Comparisons within Run B were simultaneously tested.

m Comparisons between runs (A vs B) were simultaneously tested. Differences from baseline (ie, checkpoint 1) were calculated first and then compared.

### Qualitative Emotional Experiences

Almost all (91/99, 92%) participants confirmed feeling stressed during the VR simulation, and over one-third spontaneously reported feeling anxious and/or nervous (Table 5). Six participants (3 males and 3 females) explicitly reported not feeling any stress, and 2 indicated not feeling any anxiety. The majority of participants experienced anger and/or guilt during the VR session (Table 5), with many respondents highlighting specific feelings of frustration (32/99, 31%) or annoyance (35/99, 35%). Conversely, 16 participants reported no feelings of anger, and over one quarter (27/99, 27%) indicated no feelings of guilt. Shame and betrayal were also prevalent during the simulation (>50/99, >50%), but did not linger afterward. Feelings of sadness and/or distress throughout the simulation were reported in a minority of cases, and 5% (5/99) indicated feeling overwhelmed. Two participants explicitly reported feeling morally distressed within the VR scenario, and one person reported being morally conflicted post-VR.

Once removed from the VR environment, most (59/99, 60%) participants felt “fine,” “good,” or “back to baseline.” Nearly all negative emotions appeared to resolve (Table 5), with only 12% (12/99) continuing to experience stress and ≤5% (≤5/99) indicating persistent anger, guilt, sadness, distress, or feeling overwhelmed post-VR. Similar reductions in anxiety, frustration, and annoyance were observed.

Participants also spontaneously reported experiencing several other emotions during the VR simulation (but not post-VR), including feeling: abandoned or alone (9/99, 9%), fear (9/99, 9%), sympathy or empathy (7/99, 7%), irritation (7/99, 7%), panic (6/99, 6%), torn or conflicted (4/99, 4%), burdened by the situation (1/99, 1%), and/or burdensome to others (1/99, 1%).

**Table 5.** Probed and spontaneously reported feelings and emotions experienced during the VR^a^ session (N=99).

Emotions experienced	In-VR, n (%)	Post-VR, n (%)
Stress	91 (92)	12 (12)
Anger	77 (78)	3 (3)
Guilt	68 (69)	3 (3)
Betrayal	53 (54)	0 (0)
Shame	50 (51)	0 (0)
Anxiety^b^	36 (36)	9 (9)
Annoyance^b^	35 (35)	0 (0)
Frustration^b^	32 (31)	12 (12)
Good, fine, or back to baseline	—^c^	59 (60)
Sadness or upset^b^	16 (16)	5 (5)
Distress^b^	10 (10)	4 (4)
Overwhelmed^b^	5 (5)	3 (3)
Moral distress^b^	2 (2)	1 (1)

a VR: virtual reality.

b These emotions were not explicitly asked or probed by the interviewer but were spontaneously reported by respondents.

c Not applicable.

### Familiarity With Psychoeducational Intervention Content

During the VR debrief interview, the vast majority (72/99, 73%) of participants indicated they had preexisting knowledge of all 3 stress-reduction techniques (ie, grounding, unburdening, and self-compassion) before participating in this trial. An additional 4 participants reported prior familiarity with the 3 concepts, although the terminology used in the psychoeducational intervention video was new. Furthermore, 19% (19/99) of HCWs had prior knowledge of 2 of the stress-reduction techniques presented, with the intervention introducing only one new concept: unburdening (10/99, 10%), self-compassion (7/99, 7%), or grounding (2/99, 2%). Only 2% (2/99) of HCWs reported that all 3 concepts were completely novel to them, and 2% (2/99) were newly introduced to unburdening and self-compassion but had existing awareness of grounding.

### Digital Health Monitoring

**Figure 5.**
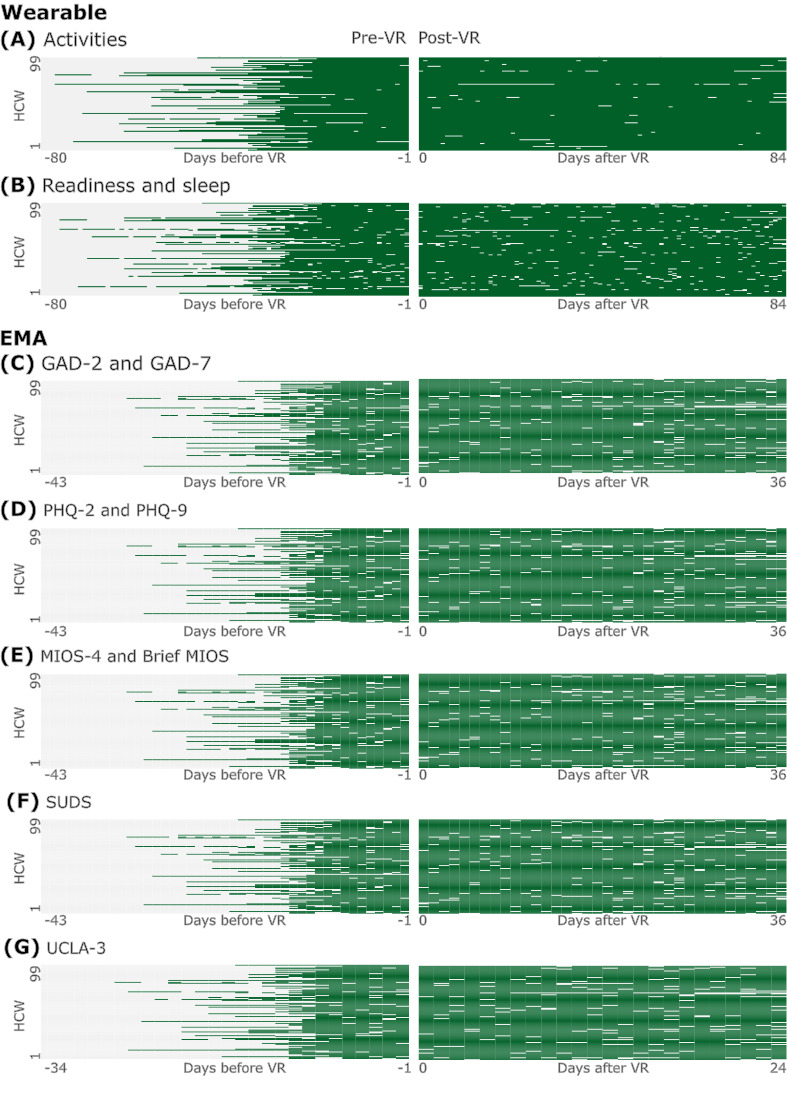
Passive wearable and ecological momentary assessment data completion indicated by green. Brief MIOS: Brief Moral Injury Outcome Scale; EMA: ecological momentary assessment; GAD-2: 2-item General Anxiety Disorder; GAD-7: 7-item General Anxiety Disorder; HCW: health care worker; MIOS-4: 4-item Moral Injury Outcome Scale; PHQ-2: 2-item Patient Health Questionnaire; PHQ-9: 9-item Patient Health Questionnaire; SUDS: Subjective Units of Distress Scale; UCLA-3: UCLA 3-item Loneliness Scale; VR: virtual reality.

Digital health monitoring began upon enrollment. The presence and participant adherence with wearable data collection from the time of screening as early as 11 weeks before VR to program completion as illustrated in Figure 5. Panels A and B collected pre-VR (days −80 to −1) and post-VR (days 1 to 84), and EMA data collection: panels C-G reported pre-VR surveys (days −43 and −34 to −1) and post-VR surveys (days 1 to 36/24, with HCWs from days 1 to 99). (A) Wearable activity data adherence pre- (left) and 12 weeks post (right) session. (B) Wearable readiness and sleep data adherence pre- (left) and 12 weeks post (right) session. Readiness data availability follows sleep data and thus the two share the same data completeness. (C) GAD-2 and GAD-7 data adherence pre- (left) and post- (right) VR session. (D) PHQ-2 and PHQ-9 data adherence pre- (left) and post- (right) VR session. (E) MIOS-4 and Brief MIOS data adherence pre- (left) and post- (right) VR session. (F) SUDS data adherence pre- (left) and post- (right) VR session. (G) UCLA-3 data adherence pre- (left) and post- (right) VR session.

Overall, high (>90%) data completion rates (Figure 5 and Table 6; indicating adherence to digital health monitoring) were observed across the study period for wearable and EMA data. Average wearable device adherence was higher in the post-VR intervention compared to pre-VR. Despite not being as high as wearable device adherence, EMA data completion also remained >90% across the study period. An increase in short-form EMA adherence was observed post intervention relative to preintervention, whereas adherence decreased post intervention for extended EMAs. Of note, once participants started a short-form or extended EMA, most items and scales were completed (ie, minimal data loss to incomplete EMAs). Rather, data loss was attributed to an absence of data for all items and scales (all-or-none). The majority of participants (70/99, 71%) completed all 12-week compliance requirements and thus received the maximum reimbursement amount. All HCWs were eligible to keep their wearable devices at the end of the study; all but 3 participants opted to keep their devices (97%) for continued personal use. The MCAR results indicated that the random nature of the missing data cannot be rejected as *P*>.22, with the exception of the readiness and sleep (*P*<.001) from the wearable and GAD-2 (*P*=.003) from the Brief EMA data. A complete MCAR result table is provided in Table S11 in Multimedia Appendix 1.

**Table 6.** Data completion percentage for wearable device features (activity, sleep, and readiness) and EMA^a^ (N=99).

Data	Pre-VR^b^ complete, mean% (SD)	Post-VR complete, mean% (SD)
Wearable		
Activity	98.59 (4.47)	98.24 (5.64)
Readiness^c^ and sleep	91.54 (10.28)	95.04 (7.63)
Short-form EMAs (biweekly)		
GAD-2^d^	91.84 (9.41)	93.43 (10.07)
PHQ-2^e^	91.79 (9.36)	93.14 (10.59)
MIOS-4^f^	91.84 (9.41)	93.14 (10.50)
SUDS^g^	91.92 (9.31)	92.63 (10.44)
UCLA-3^h^	91.67 (9.56)	92.51 (10.65)
Extended EMAs (weekly)		
GAD-7^i^	93.16 (11.13)	92.59 (13.57)
PHQ-9^j^	93.42 (11.11)	92.34 (13.56)
Brief MIOS^k^	93.42 (11.11)	92.68 (13.11)
SUDS	93.43 (11.11)	92.68 (13.11)

a EMA: ecological momentary assessment.

b VR: virtual reality.

c Readiness features depend on sleep parameters; therefore, they have the same data completeness rates (no sleep data=no readiness score).

d GAD-2: 2-item General Anxiety Disorder.

e PHQ-2: 2-item Patient Health Questionnaire.

f MIOS-4: 4-item Moral Injury Outcome Scale.

g SUDS: Subjective Units of Distress Scale.

h UCLA-3: UCLA 3-item Loneliness Scale.

i GAD-7: 7-item General Anxiety Disorder.

j PHQ-9: 9-item Patient Health Questionnaire.

k Brief MIOS: Brief Moral Injury Outcome Scale.

### Pre- to Postintervention Changes in Mental Health Symptoms

Weekly extended self-reports of GAD-7 (Δmean −0.53, SD 2.34, *t*_94_=−2.19, 95% CI −1.00 to −0.05; Cohen *d*=0.12; *P*=.03) and SUDS (Δmean −3.05, SD 11.35, *t*_94_=–2.62, 95% CI −5.37 to −0.74; Cohen *d*=0.20; *P*=.01) were significantly decreased in the 2 weeks post intervention relative to 2 weeks preintervention but were not maintained across the 12-week follow-up period (Figure S1 in Multimedia Appendix 1). Biweekly short-form EMAs showed no statistically significant changes in mental health symptoms post intervention (Table 7). The RCI results indicated that the majority of the participants had no reliable change 2 weeks pre- and post intervention. However, more participants have benefited from the intervention as indicated by the reduction in scales. With the exception of biweekly MIOS-4 (decreased =0, increased =2) and UCLA-3 (decreased =1, increased =1), there were more counts in reduced scales (indicating improvement) than in increased scales. Weekly SUDS obtained the top reduction among the weekly scale (decreased =5, increased =2). SUDS also topped the biweekly short-form, with 7 decreases and 2 increases in scale. A summary of the reduced and increased counts is provided in Table 7.

**Table 7.** Statistical summary of mental health measures 2 weeks pre- and post-VR^a^.

Data	Pre-VR, mean (SD)	Post-VR, mean (SD)	Post-Pre, Δmean (SD)	*t* test			Effect size (Cohen *d*)	RCI^b^	
				*t* test (*df*)	95% CI	*P *value		Dec.^c^	Inc.^d^
Brief EMAs^e^ (N=99)									
GAD-2^f^	1.591 (1.194)	1.459 (0.968)	−0.131 (0.794)	−1.646	−0.290 to 0.027	.10	0.121	3	0
PHQ-2^g^	1.030 (1.020)	1.055 (1.077)	0.02 (0.60)	0.402	−0.096 to 0.145	.69	0.023	2	0
MIOS-4^h^	4.007 (3.025)	4.088 (3.010)	0.081 (1.402)	0.574	−0.199 to 0.360	.57	0.027	0	2
SUDS^i^	28.813 (18.424)	27.391 (14.876)	−1.423 (12.923)	−1.095	−4.000 to 1.155	.28	0.085	7	2
UCLA-3^j^	5.015 (1.736)	4.988 (1.624)	−0.027 (0.767)	−0.349	−0.180 to 0.126	.73	0.016	1	1
Extended EMAs (n=95)^k^									
GAD-7^l^	5.057 (4.057)	4.588 (3.435)	−0.526 (2.342)	−2.190	−1.003 to −0.049	.03^o^	0.125	4	1
PHQ-9^m^	5.031 (4.206)	4.804 (4.000)	−0.321 (2.032)	−1.540	−0.735 to 0.093	.13	0.055	2	0
Brief MIOS^n^	16.289 (10.003)	15.041 (9.824)	−1.005 (5.611)	−1.746	−2.148 to 0.138	.08	0.126	4	1
SUDS	28.402 (17.949)	25.000 (15.529)	−3.053 (11.353)	−2.621	−5.365 to −0.740	.01^o^	0.203	5	0

a VR: virtual reality.

b RCI: Reliable Change Index.

c Dec.: decreased in scale indicating improvement.

d Inc.: increased in scale indicating worsening.

e EMA: ecological momentary assessment.

f GAD-2: 2-item General Anxiety Disorder.

g PHQ-2: 2-item Patient Health Questionnaire.

h MIOS-4: 4-item Moral Injury Outcome Scale.

i SUDS: Subjective Units of Distress Scale.

j UCLA-3: UCLA 3-item Loneliness Scale.

k Extended EMA has only 95 participants as 2 participants are missing 2 weeks Pre-VR and 2 missing Post-VR data.

l GAD-7: 7-item General Anxiety Disorder.

m PHQ-9: 9-item Patient Health Questionnaire.

n Brief MIOS: Brief Moral Injury Outcomes Scale.

o The differences are significant.

### Pre-Post Comparison in Passive Physiological Features (Wearable Devices)

The pre-post comparison for 70 features (29 activity, 9 readiness, and 32 sleep features) captured by the wearable was analyzed. Using false discovery rate (FDR) for family-wise error correction, no significant changes were found in the wearable features except for a percentage increase in sleep restlessness (Δmean 2.46, SD 5.43, *t*_98_=4.50; Cohen *d*=0.32; *P_adj_*<.001), indicating that participants exhibited increased movement during sleep. A complete list is provided in Tables S2-S4 in Multimedia Appendix 1.

### Correlation Between Pre-Post Changes in Wearable Features and Mental Health

**Figure 6.**
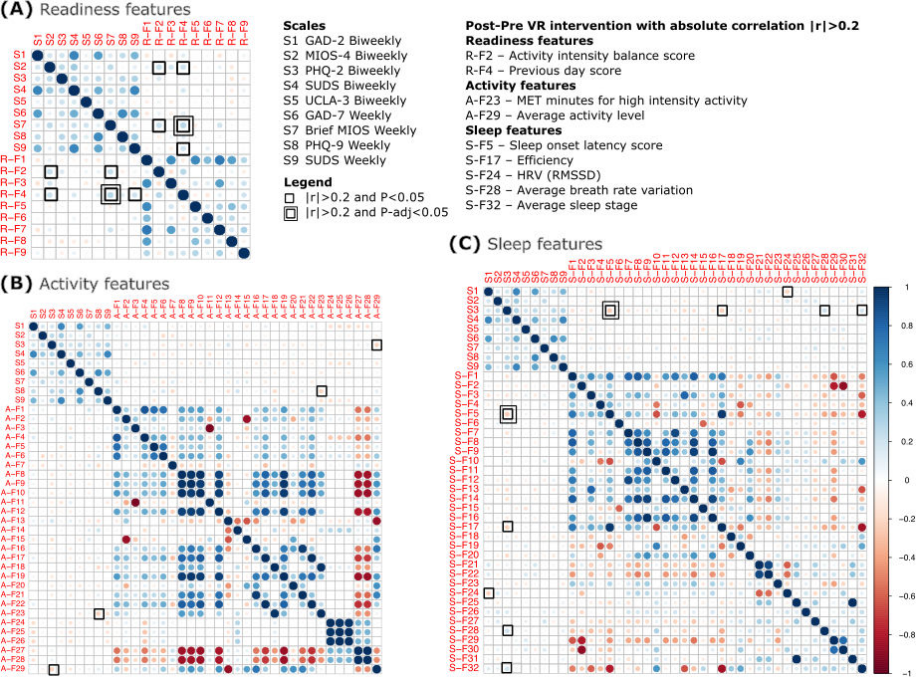
Correlation heatmap between changes in wearable features (F) and changes in scales (S1-S9 listed on top) from virtual reality. Brief MIOS: Brief Moral Injury Outcome Scale; GAD-2: 2-item General Anxiety Disorder; GAD-7: 7-item General Anxiety Disorder; HRV: heart rate variability; MIOS-4: 4-item Moral Injury Outcome Scale; PHQ-2: 2-item Patient Health Questionnaire; PHQ-9: 9-item Patient Health Questionnaire; RMSSD: root-mean-square of successive differences; SUDS: Subjective Units of Distress Scale; UCLA-3: UCLA 3-item Loneliness Scale; VR: virtual reality.

Although there were no significant changes in most of the wearable features from the intervention using FDR for family-wise error correction, changes in some features were found to have a weak correlation (0.20<|*r*|<0.30) with changes in scales. Figure 6 illustrates the correlation results between the 9 scales and the 3 sets of wearable features (R: readiness, A: activity, and S: sleep). (A) Correlation of all scales and readiness features. (B) Correlation of all scales and activity features. (C) Correlation of all scales and sleep features. Black squares indicate feature-scale correlations of |*r*|>0.20 with significant unadjusted *P* values (listed top-right) and summarized on the top right and in Table 8. Significant *P* adjusted (*P*_adj_) correlations after FDR family-wise error correction are double boxed.

The complete correlation matrix can be found in Tables S6-S8 in Multimedia Appendix 1. Changes (post-pre) in 9 features were found to be correlated (|*r*|>0.20) to changes in 6 scales, although only 2 were significant: previous day’s movement score (correlated with change in Brief MIOS: *r*=0.28, *P_adj_*=.03, 95% CI 0.08‐0.46) and sleep efficiency score (correlated with change in PHQ-2: *r*=−0.25, *P_adj_*=.04, 95% CI −0.43‐−0.06]). Table 8 also provides the mean and SD of the change, as well as the RCI results. A complete RCI analysis on the number of participants with increased or decreased value in the 70 features is provided in Tables S8-S10 in Multimedia Appendix 1.

**Table 8.** Correlations |*r*|>0.20 between changes in wearable features and EMAs^a^ pre- and post-VR^b^ intervention. The complete set of correlations is presented in Tables S4-S6 in Multimedia Appendix 1.

Feature	Post−pre Δmean (SD)	RCI^c^ Inc.^d^	RCI Dec.^e^	Scale	Pearson correlation (*r*)	95% CI	*P* value	*P*_adj_ ^f^
Readiness features								
R-F2: activity intensity balance score	0.372 (9.165)	3	4					
				MIOS-4^g^	0.237	0.041 to 0.415	.02	.07
				Brief MIOS^h^	0.230	0.030 to 0.413	.03	.09
R-F4: previous day score (movement score)^i^	−0.254 (7.879)	2	2					
				MIOS-4	0.212	0.014 to 0.394	.04	.13
				Brief MIOS^j^	0.279	0.081 to 0.456	.01	.03^j^
				SUDS^k^-weekly	0.205	0.002 to 0.391	.05	.16
Activity features								
A-F23: MET^l^ minutes for high intensity activity	5.525 (34.654)	7	4	PHQ-9^m^	−0.220	−0.404 to −0.020	.03	.09
A-F29: average activity level^n^	0.038 (0.138)	4	0	PHQ-2^o^	−0.216	−0.396 to −0.019	.03	.09
Sleep features								
S-F5: sleep efficiency score	0.714 (8.466)	9	2	PHQ-2^j^	−0.252	−0.428 to −0.058	.01	.04^j^
S-F17: efficiency	0.201 (4.540)	8	3	PHQ-2	−0.233	−0.412 to −0.037	.02	.07
S-F24: HRV^p^ (RMSSD^q^)	0.238 (7.606)	1	0	GAD-2^r^	−0.219	−0.399 to −0.023	.03	.10
S-F28: average breath rate variation	0.011 (0.235)	1	0	PHQ-2	0.219	0.022‐0.399	.03	.10
S-F32: average sleep stage^s^	−0.001 (0.100)	3	5	PHQ-2	0.200	0.004‐0.383	.05	.14

a EMA: ecological momentary assessment.

b VR:virtual reality.

c RCI: Reliable Change Index.

d Inc.: increased.

e Dec.: decreased.

f *P*_adj_ = *P* values adjusted with FDR for family-wise error correction.

g MIOS-4: 4-item Moral Injury Outcome Scale.

h Brief MIOS: Brief Moral Injury Outcome Scale.

i Previous day score is calculated using a combination of the amount of sedentary time (inactive time), vigorous activity (high output activity), and walking equivalency (measuring the total amount of activity).

j Significant correlations.

k SUDS: Subjective Units of Distress Scale.

l MET: Metabolic Equivalent of Task

m PHQ-9: 9-item Patient Health Questionnaire.

n Average activity level ranged 0‐5 with 5 being the highest activity level (recorded every 5 min).

o PHQ-2: 2-item Patient Health Questionnaire.

p HRV: heart rate variability.

q RMSSD: root-mean-square of successive differences.

r GAD-2: 2-item General Anxiety Disorder.

s Average sleep stage range 1‐4 with 1 being deep sleep and 4 being awake (recorded every 5 min).

### Correlation Between In-VR and Real-Life Stress Indicators

Taking the average of each participant’s in-VR SUDS and MIOS-4 scores, a correlation analysis was conducted with each participant’s 12-week post-VR averages of the 9 scales with Bonferroni family-wise error correction. We found a significant weak to moderate correlation between the in-VR and real-life scales. In-VR SUDS (SUDS-biweekly, SUDS-weekly, MIOS-4, Brief-MIOS: *r*=0.57, 0.58, 0.39, 0.44, all *P*<.01) and in-MIOS-4 (*r*=0.43, 0.40, 0.61, 0.58, all *P*<.009) were correlated with real-life scales, but none of the in-VR HRV comparisons were significantly correlated with real-life scales (all *P*>.20).

## Discussion

### Support of Original Hypotheses

This single-arm prospective cohort study evaluated the feasibility, engagement, and preliminary effectiveness of the DHMI-S in frontline nurses, presenting the first trial of its kind to pilot a multimodal digital suite designed to elicit and monitor stress in a virtual simulation, teach stress-reduction techniques, and continuously monitor stress through wearables and EMAs in the real world. In line with hypothesis 1a, the VR simulation successfully elicited significant increases in subjective stress (SUDS and MIOS-4), although corresponding physiological changes in HRV were minimal. Consistent with hypothesis 1b, subjective stress reactivity was attenuated during the second VR run following psychoeducation, but physiological indices again showed limited change. Partial support was observed for hypothesis 1c, as small reductions in weekly stress and anxiety were detected at 2 weeks post intervention but were not sustained at 12 weeks. Supporting hypothesis 2a, in-VR subjective stress correlated with longitudinal real-world stress measures, whereas HRV did not. Hypothesis 2b received limited exploratory support, with only weak associations between select wearable features and mental health outcomes. Finally, hypothesis 2c was supported, as high adherence was maintained across EMA and wearable monitoring. Collectively, these findings demonstrate the feasibility and short-term subjective responsiveness of the DHMI-S, while highlighting limited physiological signal detection and the need for controlled trials to establish sustained efficacy.

Secondary analyses, including RCI calculations and exploratory correlation matrices across 70 wearable-derived features and multiple EMA outcomes, provided a limited but informative signal detection. RCI results indicated that a subset of participants demonstrated reliable short-term improvement in subjective stress immediately following psychoeducation, with minimal evidence of reliable worsening. Exploratory wearable analyses involved a high volume of comparisons across activity, readiness, and sleep domains; although FDR correction was applied, the dimensionality of these analyses increases the risk of both type I and type II error. Accordingly, statistically significant wearable correlations should be interpreted as hypothesis-generating rather than confirmatory, and null findings should not be interpreted as definitive evidence of absence. Substantively, these exploratory results suggest that subjective stress responses were more sensitive to change than passive physiological metrics within this feasibility framework.

### Similarity of Results

Our findings are broadly consistent with prior work demonstrating that VR can reliably elicit subjective stress responses in controlled environments. Similar to experimental and clinical VR studies, including meta-analytic evidence for VR exposure therapy in anxiety-related conditions (eg, study by Emmelkamp and Meyerbröker [26] and Carl et al [27]), participants in our trial exhibited significant increases in self-reported distress (SUDS and MIOS-4) during the simulation, supporting the ecological validity of immersive stress paradigms. The attenuation of subjective stress during the second VR run is consistent with habituation and inhibitory learning models observed in repeated VR exposure contexts [27] and aligns with research demonstrating that VR can serve as a controlled platform for both stress induction and emotion regulation training [22]. However, unlike some VR-based stress-reduction trials reporting physiological modulation—such as HRV changes during acute stress or relaxation phases [28]—our study observed minimal and inconsistent HRV effects. This was surprising given the established relationship between stress and decreases in HRV [54]. As HRV-RMSSD has traditionally been estimated using a 5-minute window time [55], other acute measures of stress may have been more appropriate in a 10-minute VR simulation. This discrepancy may relate to the short simulation duration [56], the use of RMSSD with 5-minute overlapping windows [18], or the brief psychoeducational dose delivered, compared to multisession interventions.

The short-term reductions in weekly stress and anxiety at 2 weeks post intervention are consistent with meta-analyses showing that mindfulness-based and psychoeducational interventions yield small but significant improvements in HCWs’ mental health [10,57]. However, the absence of sustained effects at 12 weeks mirrors broader digital mental health findings, where symptom improvements often diminish without booster sessions or ongoing engagement [21]. With respect to passive physiological monitoring, our limited wearable findings align with recent reviews indicating small effect sizes and mixed validity for consumer-grade devices, especially the Oura Ring, in mental health research [19,24]. The weak correlations between wearable features and symptom change in our study are therefore consistent with the growing recognition that some commercial metrics lack specificity for psychological stress as effect size in psychiatry research is generally small [58,59].

A prominent challenge surrounding digital mental health interventions is promoting participant engagement [32], especially for trials with long-term (>2 wk) follow-up periods, as reflected in our pilot study with <50% data completion [33]. Notably, our high adherence rates (>90%) substantially exceed those reported in large-scale remote digital health studies, where median retention ranges from days to a few weeks [32] and where attrition in mobile mental health trials is common [31]. This suggests that structured engagement strategies, such as combining reminders, human follow-up, and compensation, may meaningfully enhance retention compared to typical digital intervention benchmarks. Existing literature suggests that monetary incentives reliably increase short-term adherence, while human follow-up increases retention for technically complex protocols [32,60,61]. A large study with over 1000 participants with small monetary rewards found that participant payment was not enough to maintain participant engagement [31]. Therefore, high adherence in this study likely reflects a synergistic effect of these components and may provide an overestimation of adherence in routine care that may not have comparable resources. Interestingly, adherence was marginally higher for extended weekly assessments than for biweekly assessments, warranting further investigation into the nature of these adherence differences (eg, conflicts with work schedules and perceived redundancy) and considerations for future DHMI-S deployments. Future studies should quantify the contribution of each engagement strategy and report economical and feasible alternatives for real-world clinical integration.

Collectively, our results converge with prior literature in demonstrating robust subjective stress induction, modest short-term symptom improvement, and feasibility of VR-based digital monitoring, while diverging in the limited physiological signal detection and lack of sustained effects—highlighting both the promise and current limitations of integrated multimodal digital stress platforms.

### Interpretation

The present findings should be interpreted within the context of several methodological constraints affecting internal, statistical, and measurement validity. First, the single-arm prospective cohort design precludes causal inference. In the absence of a randomized comparator, reductions in subjective stress between VR runs cannot be definitively attributed to the psychoeducational intervention. Alternative explanations include habituation to the simulated scenario, expectancy effects, regression to the mean, demand characteristics [62], or desensitization due to repeated exposure [5]. Although repeated exposure is itself consistent with mechanisms underlying VR exposure therapy [27], the design does not allow isolation of psychoeducation-specific effects from repetition effects. Repeated exposure to stressful VR scenarios could serve as a meaningful way to mitigate negative emotional responses to one’s environment and reduce stress [27]. Since memory is context-dependent, practicing stress-reduction strategies immediately after the intervention in a VR environment may also enhance learning [63], improving people’s ability to apply these skills in real-life scenarios. In this sense, VR could be administered preemptively as a training tool (ie, a “digital vaccine”) or as a post hoc treatment [25] for existing stress. As such, the attenuation observed in Run B should be considered preliminary and hypothesis-generating rather than confirmatory.

Second, measurement precision varied across modalities. Subjective stress scales (SUDS and MIOS-4) are sensitive to transient affective shifts but remain susceptible to reporting bias and shared-method variance [64,65], particularly when correlating conceptually overlapping constructs across contexts. The significant correlations between in-VR and longitudinal real-world stress measures may partially reflect trait-level stress reactivity or scale similarity rather than predictive validity per se. It is not surprising that comparisons of scores on similar or identical scales would yield significant correlations; those who are more prone to stress in one context are likely prone to stress in another due to the individual stress process [66]. However, the fact that in-VR stress correlates with real-world stress further supports the finding that the VR simulation elicited stress [67,68]. These results provide proof of concept for the use of VR for identifying those who are at greatest risk of stress in real-world contexts (eg, military and health care), which could enable proactive intervention among those deemed to be at higher risk. In the 2 weeks after the VR session, acceptable improvements for initial feasibility were observed in the weekly measurements of stress (SUDS) and anxiety (GAD-7), consistent with an intervention effect on participant mental health. However, these effects diminished before the end of the 12-week monitoring period, suggesting a need for a more powerful intervention or more sessions to maintain benefits [40].

Physiological measurement also carries imprecision. HRV (RMSSD) estimation using overlapping 5-minute windows during a 10-minute VR simulation may have limited sensitivity to rapid autonomic fluctuations. Furthermore, consumer-grade wearable features (70 in total) vary in validation status and clinical interpretability. The dimensionality of these features reduces statistical power after correction for multiple comparisons and increases the likelihood of both type I and type II errors. Although FDR procedures were applied, the overall number of tests and overlap among wearable-derived metrics constrain interpretability, and null findings should not be interpreted as definitive evidence of the absence of physiological change.

Third, statistical validity is influenced by the volume and structure of analyses. The study examined multiple outcomes (subjective, physiological, EMA-based, and wearable-derived), multiple checkpoints, and multiple timeframes (acute, 2-wk, and 12-wk). While adjustments were applied where appropriate, the analytic breadth increases the risk of spurious associations and may dilute power for detecting small but meaningful effects. Reported wearable effect sizes fall within ranges commonly observed in ambulatory digital health research [58], but may reflect limited precision or insufficient dosing (such as refresher courses [69]) rather than the true absence of physiological modulation. Sampling considerations also affect generalizability and inference [70]. The sample size (N=99) was adequate for feasibility evaluation and within-participant comparisons but was not powered for small physiological effect detection across high-dimensional wearable features.

Finally, the qualitative content analysis findings should be interpreted within the constraints of a structured postsimulation debrief and deductive coding framework. Although interrater reliability was high, emotion coding relied on participant self-report and researcher interpretation of brief narrative responses, which may underestimate nuanced or ambivalent affective states. The high frequency of stress-, anger-, guilt-, and shame-related responses supports the construct validity of the VR scenario as a morally and emotionally evocative stimulus; however, rapid post-VR emotional resolution reported by most participants may reflect demand characteristics [62], social desirability bias [71], or the immediate psychological containment provided by structured debriefing (PEARLS framework [47]). Additionally, the binary presence-absence coding approach prioritized reliability over thematic depth and may not fully capture intensity, complexity, or moral residue processes described in the broader moral distress literature. Accordingly, these qualitative findings provide supportive but not exhaustive evidence of emotional engagement and psychological safety within the simulation context. Future planned manuscripts will delve deeper into the underlying themes and recurring patterns related to emotions and cognition in-VR, clinical decision-making, real-world experiences of moral distress, and user feedback on the DHMI-S to inform these central elements further.

Taken together, these findings provide preliminary support for feasibility, stress induction validity, short-term subjective responsiveness, and emotional safety within a multimodal digital stress platform. However, threats to causal inference, measurement imprecision—particularly in physiological metrics—multiple testing burden, and sampling constraints necessitate cautious interpretation. Future randomized or hybrid effectiveness-implementation trials with prespecified physiological end points, reduced feature dimensionality, optimized dosing schedules, and diverse samples will be required to establish mechanism, durability, and clinical efficacy.

### Limitations

The methodological framework of this study has certain limitations that could be addressed in future research. Primarily, interpretation of our findings is limited by the absence of a randomized comparator group and constrains our ability to establish causal relationships. However, it is important to acknowledge that this study was designed to evaluate feasibility, adherence, and preliminary effectiveness rather than to confirm efficacy through an RCT. Implementation science frameworks suggest a phased approach, where interventions undergo observational testing before advancing to controlled trials [40,72]. Due to the VR and psychoeducation components representing an exploratory combination rather than a standardized protocol, it is unclear what magnitude of subjective or physiological change should be expected. Moreover, pragmatic trials and stepped-wedge designs may be more appropriate than traditional RCTs for evaluating digital mental health interventions, as they better reflect real-world implementation constraints [73]. Future studies should build on these findings by incorporating comparative designs, such as hybrid effectiveness-implementation trials, to further validate the intervention’s impact while maintaining ecological validity.

Physiological indicators, including HRV, showed minimal group-level shifts, and there is no consensus on what constitutes a clinically meaningful change in these metrics for brief digital interventions. As a result, any observed changes should be viewed as preliminary signals only. It is also worth noting that the VR scenario used here was developed in relation to COVID-related moral distress; thus, generalizability to nonpandemic stressors or other high-stress settings may be limited. It is also worth acknowledging that the use of psychoeducation coping strategies was not permitted (ie, no opportunities for unburdening) or was not objectively measured during Run B. Without time-stamped indicators of when participants engaged in stress-reduction strategies, we cannot link specific components of the psychoeducational intervention to subsequent changes in subjective or physiological stress. This constrains our ability to draw conclusions about mechanisms of change. Future work should ensure participants have opportunities to practice learned strategies directly in the VR environment and should systematically track strategy use to clarify cognitive and physiological mechanisms. Possible approaches include embedding brief, required practice trials that generate time-stamped markers, using real-time respiration monitoring to detect engagement in diaphragmatic breathing, or incorporating immediate ecological momentary prompts following stressful events.

Second, the absence of maintained intervention effects at 12 weeks post intervention raises concerns around administration dosage (timing and frequency) and protocols for optimal stress management. It is also important to consider the wording used in frequent EMAs. Specifically, this study did not modify the validated wording for the weekly and biweekly EMAs (referenced to a 2-wk time period), despite administration at a higher frequency. Thus, the inability to detect long-term changes in mental health outcomes post intervention may be limited by insensitivity to short-term symptom changes (daily, biweekly, and weekly). Emerging evidence supports the need to modify EMA wording in accordance with the administration timeframe, including recent validation of PHQ-9 and GAD-7 metrics [74].

Third, the dependence on stratified analytical approaches, though effective for capturing preintervention and group-level trends, may oversimplify the dynamic and latent factors influencing the effects of the intervention. Advanced multilevel modeling and time-series modeling techniques, such as cluster-based or growth-trajectory models, could provide a more nuanced understanding of variations and the longitudinal impact of the intervention. Incorporating these advanced analytical techniques would allow for a more thoughtful exploration of latent constructs and offer more precise recommendations.

Additionally, high adherence, while a strength, was achieved through structured engagement supports (reminders, compensation, and device-retention incentives) that may not generalize to routine care contexts.

### Conclusions

Collectively, this study demonstrates that a multimodal DHMI-S integrating VR, psychoeducation, EMAs, and wearable devices can be implemented successfully in a high-demand occupational setting and can meaningfully engage HCWs in stress monitoring and skill practice. Beyond feasibility, the findings suggest that immersive simulation may serve as both an assessment tool and a training environment, providing a structured way to identify stress vulnerability and rehearse coping strategies within contextually realistic scenarios. At the same time, the limited physiological signal detected through consumer wearables highlights an important gap between technological capabilities and clinically meaningful biomarker identification, underscoring the need for more precise digital phenotyping approaches. Although short-term improvements in subjective stress were observed, durability likely requires repeated or longitudinal intervention dosing rather than a single exposure. Taken together, these results position the DHMI-S not simply as a standalone intervention, but as a scalable framework for proactive stress surveillance and skill-based resilience training in health care and other high-risk professions. Future controlled and implementation-focused trials are warranted to refine its mechanisms, optimize delivery frequency, and determine how much multimodal digital systems can be sustainably integrated into real-world mental health care.

## Supplementary material

10.2196/77818 Multimedia Appendix 1 Extended analytical results.

10.2196/77818 Checklist 1 STROBE checklist.

## Abbreviations

AR: aligned rank
ART-ANOVA: aligned rank transform ANOVA
Brief MIOS: Brief Moral Injury Outcome Scale
DHMI-S: Digital Health Monitoring and Intervention Suite for Stress
ECG: electrocardiography
EMA: ecological momentary assessment
FDR: false discovery rate
GAD-2: 2-item Generalized Anxiety Disorder
GAD-7: 7-item Generalized Anxiety Disorder
HCW: health care worker
HRV: heart rate variability
MCAR: Missing Completely at Random
MIOS-4: 4-item Moral Injury Outcome Scale
PEARLS: Promoting Excellence and Reflective Learning in Simulation
PHQ-9: 9-item Patient Health Questionnaire
RCI: Reliable Change Index
RCT: randomized controlled trial
RMSSD: root-mean-square of successive differences
STROBE: Strengthening the Reporting of Observational Studies in Epidemiology
SUDS: Subjective Units of Distress Scale
UCLA-3: UCLA 3-item Loneliness Scale
VR: virtual reality
