# Supplementary material for: Digital Health Monitoring and Intervention Suite for Stress in Frontline Nurses: Prospective Cohort Trial
Source: JMIR Form Res. 2026 Jun 11;10:e77818. doi: 10.2196/77818 (PMC13258063; doi:10.2196/77818)
Supplement: Multimedia Appendix 1 [file formative-v10-e77818-s001.docx]

Table of Contents

[In-VR Data Mean and Standard Deviation 2](#_Toc223367747)

[EMA Data 3](#_Toc223367748)

[Changes in Wearable Data 4](#_Toc223367749)

[Correlation in Changes in EMA and Wearable 6](#_Toc223367750)

[Reliable Change Index in Wearable 8](#_Toc223367751)

[Missing Completely at Random 10](#_Toc223367752)

# In-VR Data Mean and Standard Deviation

| **Checkpoint** | **MIOS-4^1^ (*N*=99)** | **SUDS^2^** **(*N*=99)** | **HRV RMSSD (msec)^3^ (N=91)^4^** |
| --- | --- | --- | --- |
|  | ***M* (*SD*)** | ***M* (*SD*)** | ***M* (*SD*)** |
| 1A | 2·95 (3·16) | 25·05 (16·00) | 25·06 (13·04) |
| 2A | 6·42 (3·45) | 51·92 (21·17) | 24·51 (13·32) |
| 3A | 7·05 (3·61) | 53·43 (21·25) | 24·79 (13·72) |
| 4A | 7·96 (3·75) | 48·69 (21·12) | 24·83 (13·26) |
| 1B | 4·37 (3·39) | 26·57 (16·30) | 25·50 (13·25) |
| 2B | 6·74 (3·63) | 45·86 (22·22) | 26·07 (13·20) |
| 3B | 6·97 (3·78) | 46·67 (22·36) | 26·19 (13·16) |
| 4B | 6·89 (3·60) | 40·51 (21·92) | 28·83 (14·10) |
| ^1^MIOS-4 has a minimum of 0 for all checkpoints  ^2^SUDS has a minimum of 10 for all checkpoints  ^3^HRV measured in root mean square of successive differences [RMSSD] with a metric of milliseconds (msec).  ^4^Poor quality ECG signals (*n* = 3) and missing R-waves (*n* = 5) were excluded from analysis | | | |

Table S1 | In-VR descriptive statistics of subjective self-report (MIOS-4 and SUDS) and objective physiological (HRV) markers of stress across VR simulation checkpoints.

# EMA Data


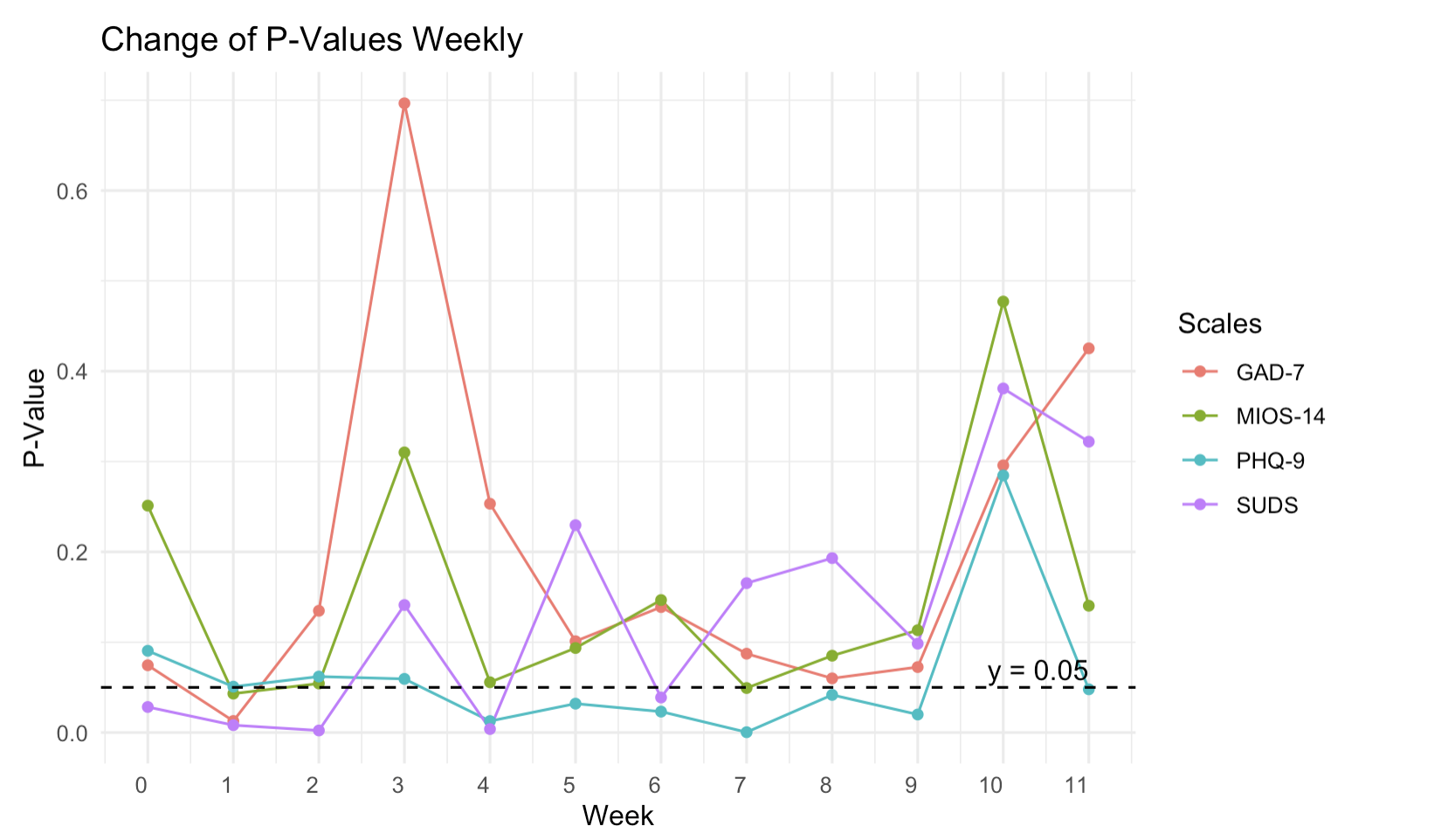


**(A)**


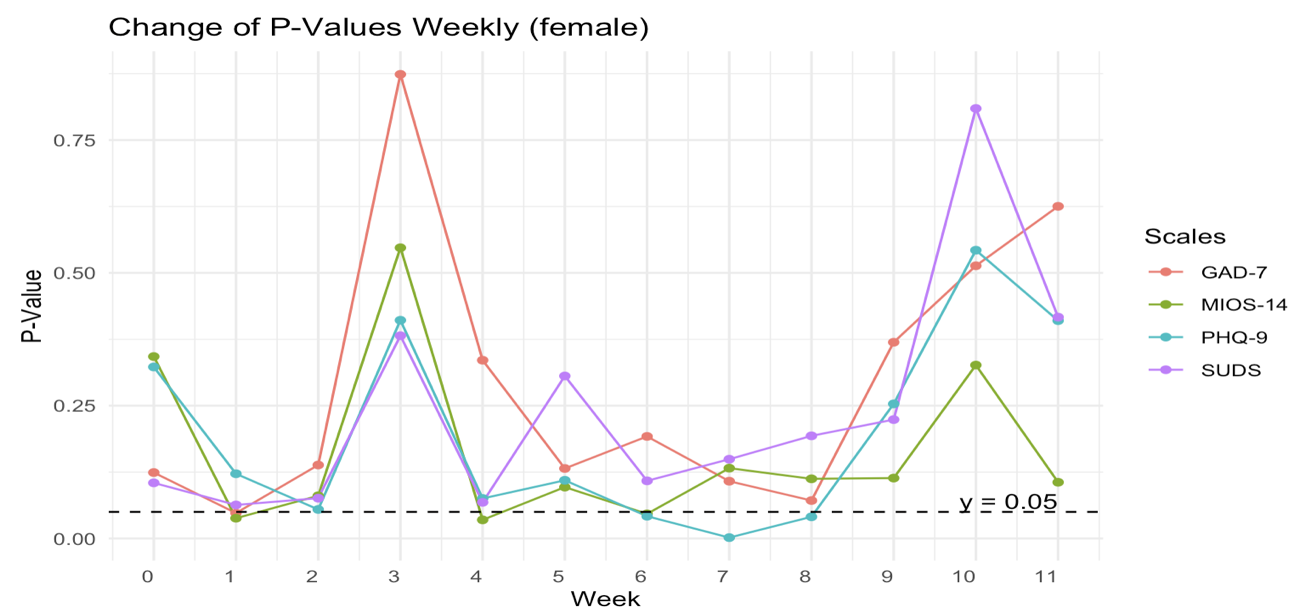


**(**B**)**


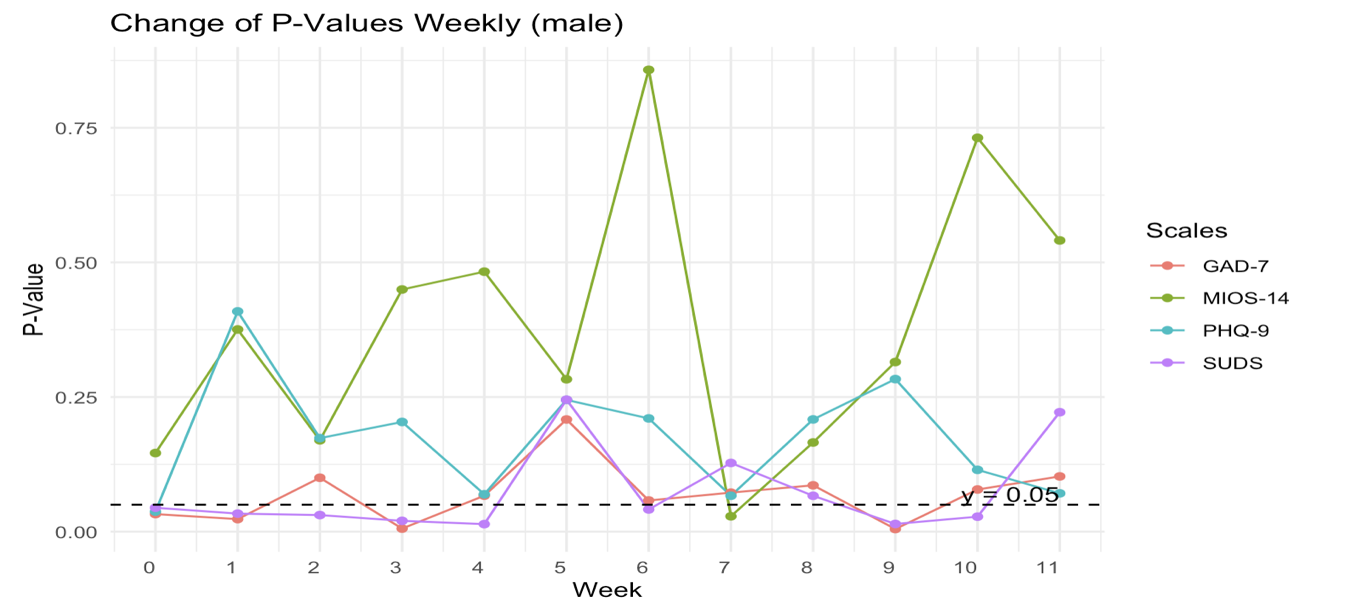


**(C)**

Figure S1 | Weekly EMA Post-VR comparing to Pre-VR intervention stratified by sex.

Out of the 99 participants, there were 86 females and 13 males. The EMA data was dominated by the female group illustrated above in the pre-post comparative study. Panel A: Comparing weekly extended EMA for all 99 participants. Panel B: The results for females only. Panel C: The results for males only.

# Changes in Wearable Data

This is a summary of changes of all physiological measure from two weeks before (-1) to two weeks after (1) the VR intervention.

1. Activity

|  | ***ΔM*** | ***t-test*** | ***DF*** | ***P-adj*** | ***Cohen’s d*** |
| --- | --- | --- | --- | --- | --- |
| F1 | 0.08 | 0.08 | 98 | 0.94 | 0.01 |
| F2 | -0.45 | -0.85 | 97 | 0.52 | 0.05 |
| F3 | -0.14 | -0.59 | 97 | 0.65 | 0.06 |
| F4 | -2.74 | -1.51 | 97 | 0.23 | 0.08 |
| F5 | 1.54 | 0.73 | 97 | 0.58 | 0.06 |
| F6 | 1.60 | 1.37 | 97 | 0.28 | 0.09 |
| F7 | 0.14 | 0.31 | 97 | 0.82 | 0.03 |
| F8 | 20.09 | 2.56 | 98 | 0.07 | 0.12 |
| F9 | 25.46 | 2.61 | 98 | 0.07 | 0.06 |
| F10 | 444.26 | 2.59 | 98 | 0.07 | 0.13 |
| F11 | 0.02 | 0.70 | 98 | 0.58 | 0.07 |
| F12 | 471.92 | 2.98 | 98 | 0.07 | 0.15 |
| F13 | -19.99 | -2.36 | 98 | 0.08 | 0.13 |
| F14 | -0.81 | -0.14 | 98 | 0.92 | 0.01 |
| F15 | 9.11 | 1.57 | 98 | 0.22 | 0.09 |
| F16 | 9.89 | 2.08 | 98 | 0.12 | 0.12 |
| F17 | 2.85 | 2.19 | 98 | 0.11 | 0.11 |
| F18 | 0.57 | 1.35 | 98 | 0.28 | 0.10 |
| F19 | 0.02 | 2.40 | 98 | 0.08 | 0.13 |
| F20 | 0.08 | 0.45 | 98 | 0.73 | 0.03 |
| F21 | 5.76 | 1.82 | 98 | 0.19 | 0.09 |
| F22 | 8.70 | 2.06 | 98 | 0.12 | 0.10 |
| F23 | 5.52 | 1.59 | 98 | 0.22 | 0.12 |
| F24 | 7.68 | 1.57 | 98 | 0.22 | 0.10 |
| F25 | 0.17 | 1.58 | 98 | 0.22 | 0.13 |
| F26 | 0.11 | 1.58 | 98 | 0.22 | 0.13 |
| F27 | -0.25 | -1.20 | 98 | 0.32 | 0.07 |
| F28 | -0.15 | -1.20 | 98 | 0.32 | 0.07 |
| F29 | 0.04 | 2.70 | 98 | 0.07 | 0.14 |

Table S2 *|* Pre-post summary of Activity features for the wearable including outliers.

1. Readiness

|  | ***ΔM*** | ***t-test*** | ***DF*** | ***P-adj*** | ***Cohen’s d*** |
| --- | --- | --- | --- | --- | --- |
| F1 | 0.42 | 0.83 | 98 | 0.82 | 0.08 |
| F2 | 0.37 | 0.40 | 98 | 0.82 | 0.03 |
| F3 | -0.27 | -0.22 | 98 | 0.82 | 0.03 |
| F4 | -0.25 | -0.32 | 97 | 0.82 | 0.02 |
| F5 | 1.05 | 1.15 | 98 | 0.82 | 0.11 |
| F6 | 0.77 | 0.67 | 98 | 0.82 | 0.07 |
| F7 | -0.29 | -0.26 | 98 | 0.82 | 0.03 |
| F8 | 1.06 | 0.98 | 98 | 0.82 | 0.08 |
| F9 | 0.64 | 0.98 | 98 | 0.82 | 0.12 |

Table S3 | Pre-post summary of Readiness features for the wearable including outliers.

1. Sleep

|  | ***ΔM*** | ***t-test*** | ***DF*** | ***P-adj*** | ***Cohen’s d*** |
| --- | --- | --- | --- | --- | --- |
| F1 | 0.90 | 1.55 | 98 | 0.44 | 0.12 |
| F2 | 3.44 | 1.98 | 98 | 0.20 | 0.15 |
| F3 | -0.16 | -0.16 | 98 | 0.97 | 0.01 |
| F4 | -0.02 | -0.03 | 98 | 0.98 | 0.00 |
| F5 | 0.71 | 0.84 | 98 | 0.90 | 0.07 |
| F6 | 1.55 | 2.37 | 98 | 0.16 | 0.21 |
| F7 | 0.96 | 0.85 | 98 | 0.90 | 0.07 |
| F8 | 0.81 | 0.88 | 98 | 0.90 | 0.09 |
| F9 | 273.13 | 0.82 | 98 | 0.90 | 0.09 |
| F10 | -17.91 | -0.14 | 98 | 0.97 | 0.01 |
| F11 | 470.17 | 2.46 | 98 | 0.16 | 0.22 |
| F12 | 12.14 | 0.11 | 98 | 0.97 | 0.01 |
| F13 | -191.27 | -2.00 | 98 | 0.20 | 0.15 |
| F14 | 291.04 | 1.02 | 98 | 0.90 | 0.11 |
| F15 | -2.23 | -0.07 | 98 | 0.97 | 0.01 |
| F16 | 121.76 | 0.73 | 98 | 0.90 | 0.08 |
| F17 | 0.20 | 0.44 | 98 | 0.92 | 0.04 |
| **F18** | **2.46** | **4.50** | **98** | **0.0006** | **0.32** |
| F19 | 0.03 | 0.47 | 98 | 0.92 | 0.04 |
| F20 | -0.07 | -0.55 | 98 | 0.92 | 0.04 |
| F21 | -0.05 | -0.20 | 98 | 0.97 | 0.01 |
| F22 | -0.11 | -0.48 | 98 | 0.92 | 0.02 |
| F23 | 168.27 | 0.58 | 98 | 0.92 | 0.05 |
| F24 | 0.24 | 0.31 | 98 | 0.97 | 0.01 |
| F25 | -0.01 | -0.71 | 98 | 0.90 | 0.10 |
| F26 | 369.11 | 0.28 | 98 | 0.97 | 0.04 |
| F27 | -0.11 | -3.02 | 98 | 0.05 | 0.07 |
| F28 | 0.01 | 0.46 | 98 | 0.92 | 0.02 |
| F29 | -1660.46 | -2.26 | 98 | 0.17 | 0.22 |
| F30 | -1257.69 | -2.07 | 98 | 0.20 | 0.20 |
| F31 | -0.01 | -0.71 | 98 | 0.90 | 0.10 |
| F32 | 0.00 | -0.14 | 98 | 0.97 | 0.01 |

Table S4 | Pre-post summary of Sleep features for the wearable including outliers.

# Correlation in Changes in EMA and Wearable

1. Activity

|  | **A-F1** | **A-F2** | **A-F3** | **A-F4** | **A-F5** | **A-F6** | **A-F7** | **A-F8** | **A-F9** | **A-F10** | **A-F11** | **A-F12** | **A-F13** | **A-F14** | **A-F15** |
| --- | --- | --- | --- | --- | --- | --- | --- | --- | --- | --- | --- | --- | --- | --- | --- |
| S1 | 0.11 | 0.11 | 0.07 | 0.16 | 0.01 | -0.02 | -0.07 | 0.08 | 0.07 | 0.08 | -0.08 | 0.04 | 0.09 | 0.02 | -0.12 |
| S2 | 0.09 | 0.01 | -0.05 | 0.05 | -0.01 | -0.18 | -0.14 | -0.04 | -0.04 | -0.03 | -0.02 | -0.04 | 0.11 | 0.04 | -0.04 |
| S3 | -0.01 | 0.15 | 0.04 | 0.03 | -0.05 | -0.13 | 0.02 | -0.12 | -0.14 | -0.11 | -0.09 | -0.11 | 0.19 | 0.03 | -0.17 |
| S4 | -0.01 | -0.05 | 0.08 | 0.03 | -0.14 | -0.10 | -0.11 | -0.05 |  | -0.05 | -0.14 | -0.06 | 0.07 | 0.05 | -0.01 |
| S5 | 0.05 | 0.00 | -0.13 | 0.13 | -0.03 | -0.09 | -0.06 | -0.11 | -0.11 | -0.10 | 0.12 | -0.09 | 0.11 | -0.03 | 0.03 |
| S6 | 0.05 | 0.08 | -0.01 | 0.00 | 0.02 | 0.03 | 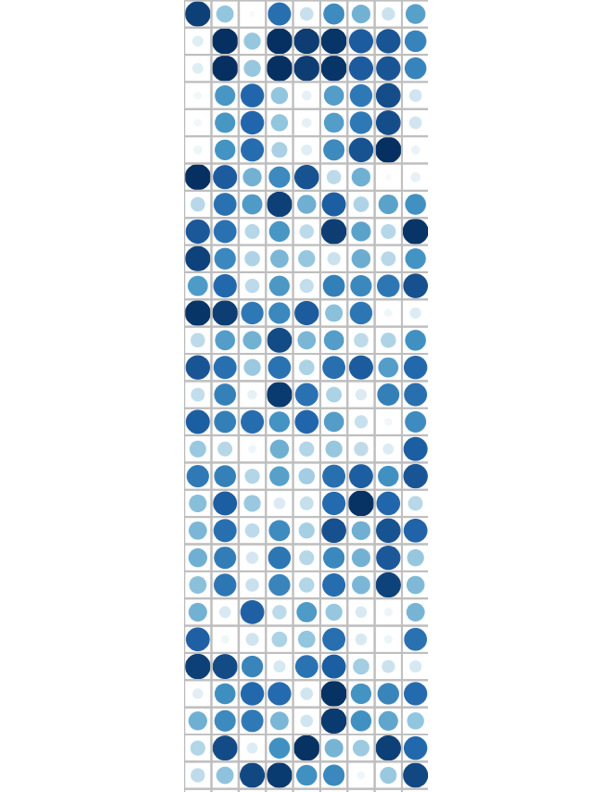-0.09 | -0.03 | -0.05 | -0.02 | 0.03 | -0.03 | 0.09 | 0.06 | -0.10 |
| S7 | 0.19 | -0.09 | -0.05 | -0.06 | -0.10 | -0.15 | 0.15 | -0.08 | -0.07 | -0.07 | 0.00 | -0.02 | -0.12 | 0.13 | 0.15 |
| S8 | 0.09 | 0.01 | 0.07 | 0.05 | 0.13 | 0.19 | -0.19 | -0.01 | -0.02 | 0.02 | -0.03 | 0.05 | -0.15 | 0.20 | 0.04 |
| S9 | 0.01 | -0.03 | 0.09 | 0.03 | -0.14 | -0.03 | -0.08 | -0.08 | -0.09 | -0.03 | -0.11 | -0.02 | -0.02 | 0.05 | -0.03 |
|  |  |  |  |  |  |  |  |  |  |  |  |  |  |  |  |
|  | **A-F16** | **A-F17** | **A-F18** | **A-F19** | **A-F20** | **A-F21** | **A-F22** | **A-F23** | **A-F24** | **A-F25** | **A-F26** | **A-F27** | **A-F28** | **A-F29** |  |
| S1 | 0.02 | 0.11 | 0.00 | 0.06 | 0.01 | 0.02 | 0.11 | 0.00 | -0.18 | -0.19 | -0.19 | -0.15 | -0.15 | -0.01 |  |
| S2 | -0.03 | -0.06 | -0.01 | -0.03 | -0.05 | -0.03 | -0.03 | 0.02 | -0.05 | -0.06 | -0.06 | 0.00 | 0.00 | -0.09 |  |
| S3 | -0.09 | -0.07 | -0.04 | -0.11 | -0.10 | -0.11 | -0.06 | -0.07 | -0.03 | -0.03 | -0.03 | 0.09 | 0.09 | **-0.22** |  |
| S4 | -0.03 | -0.01 | -0.05 | -0.06 | 0.08 | -0.05 | -0.01 | -0.05 | -0.10 | -0.09 | -0.09 | 0.00 | 0.00 | -0.03 |  |
| S5 | -0.10 | -0.08 | -0.02 | -0.12 | -0.09 | -0.11 | -0.07 | -0.02 | -0.15 | -0.16 | -0.16 | 0.01 | 0.01 | -0.12 |  |
| S6 | -0.03 | 0.06 | -0.08 | -0.04 | -0.13 | 0.01 | 0.02 | -0.12 | -0.05 | -0.06 | -0.06 | 0.00 | 0.00 | -0.05 |  |
| S7 | -0.02 | -0.12 | 0.04 | -0.05 | 0.07 | -0.07 | -0.11 | 0.07 | -0.02 | -0.04 | -0.04 | 0.02 | 0.02 | 0.07 |  |
| S8 | 0.06 | 0.11 | -0.19 | -0.04 | -0.11 | 0.11 | 0.06 | **-0.22** | 0.00 | 0.02 | 0.02 | 0.02 | 0.02 | 0.13 |  |
| S9 | -0.03 | 0.05 | -0.15 | -0.02 | -0.06 | 0.00 | 0.05 | -0.17 | -0.18 | -0.13 | 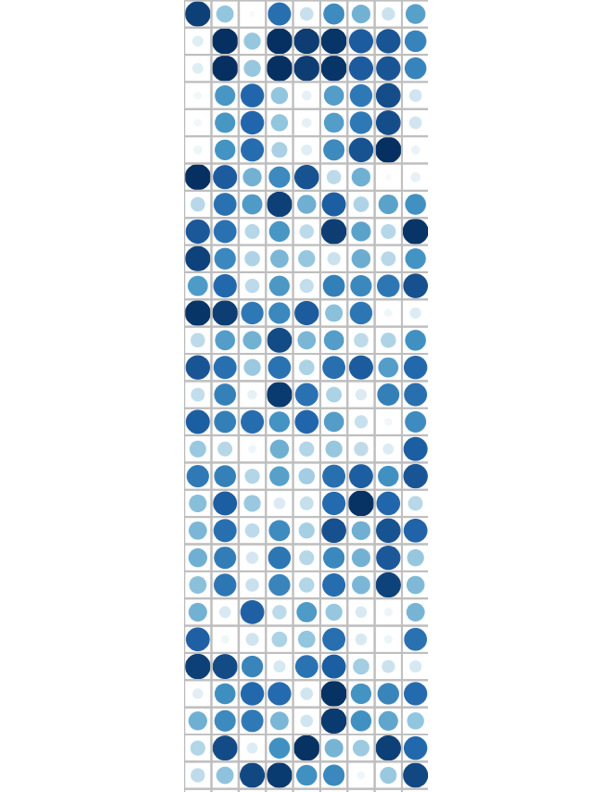-0.13 | -0.05 | -0.05 | 0.06 |  |
| **BOLD** font indicating \|r\|>.20 and *p*<0.05 (**A-F23–S8** and **A-F29–S3**).  The background ellipse illustrated the p-values (darker closer to 1 and lighter closer to 0). | | | | | | | | | | | | | | | |

Table S5 | Correlation of Post>Pre in EMA and Activity features thresholding at |r|>0.20.

1. Readiness

|  | **R-F1** | **R-F2** | **R-F3** | **R-F4** | **R-F5** | **R-F6** | **R-F7** | **R-F8** | **R-F9** |
| --- | --- | --- | --- | --- | --- | --- | --- | --- | --- |
| S1 | -0.13 | 0.04 | -0.15 | 0.12 | 0.02 | -0.04 | -0.13 | -0.02 | 0.07 |
| S2 | 0.09 | **0.24** | 0.18 | **0.21** | -0.06 | 0.04 | 0.09 | -0.11 | 0.02 |
| S3 | -0.07 | 0.15 | -0.01 | 0.03 | -0.04 | -0.03 | 0.00 | -0.13 | -0.08 |
| S4 | -0.05 | 0.04 | -0.02 | 0.17 | -0.01 | 0.07 | 0.01 | -0.10 | 0.08 |
| S5 | -0.09 | 0.08 | -0.10 | -0.03 | -0.03 | 0.04 | 0.02 | -0.11 | -0.03 |
| S6 | -0.11 | 0.09 | -0.09 | 0.12 | -0.01 | -0.02 | -0.03 | -0.08 | -0.02 |
| S7 | 0.02 | **0.23** | -0.02 | ***0*.*28*** | 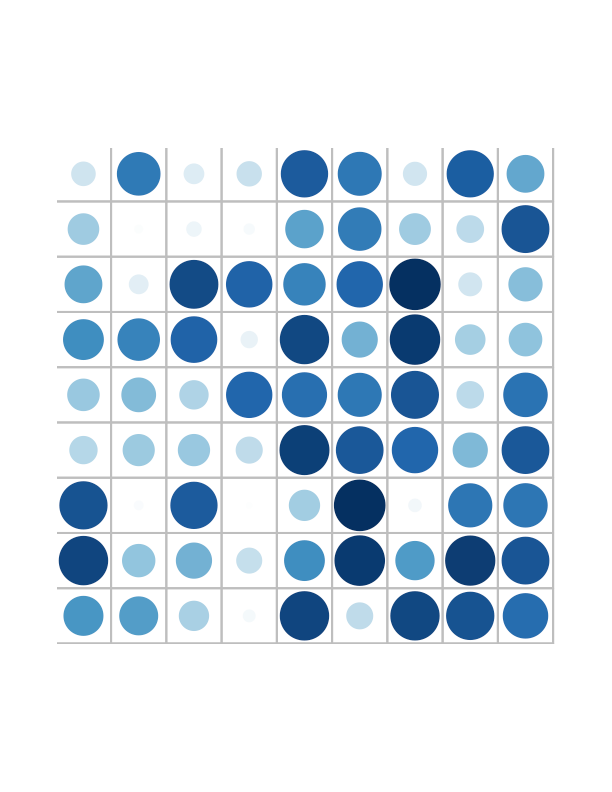-0.10 | 0.00 | 0.20 | -0.04 | 0.04 |
| S8 | -0.01 | -0.09 | 0.07 | 0.12 | 0.05 | 0.01 | 0.06 | 0.01 | 0.02 |
| S9 | -0.06 | 0.06 | -0.10 | **0.20** | -0.01 | 0.12 | 0.01 | -0.02 | 0.03 |
| **BOLD** font indicating \|r\|>.20 and *p*<0.05 (**R-F2–S2**, **R-F2–S7**, **R-F4–S2**, and **R-F4–S9**)  **BOLD** and *Italic* font indicating \|r\|>.20 and FDR p-adjusted<0.05 (***R-F4–S7***)  The background ellipse illustrated the p-values (darker closer to 1 and lighter closer to 0). | | | | | | | | | |

Table S6 | Correlation of Post>Pre in EMA and Readiness features thresholding at |r|>0.20.

1.
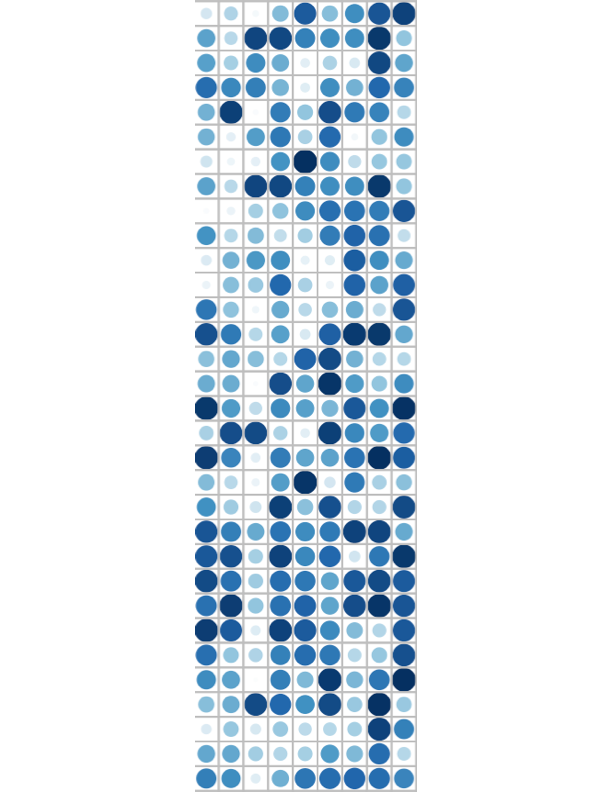
Sleep

|  | **S-F1** | **S-F2** | **S-F3** | **S-F4** | **S-F5** | **S-F6** | **S-F7** | **S-F8** | **S-F9** | **S-F10** | **S-F11** | **S-F12** | **S-F13** | **S-F14** | **S-F15** | **S-F16** |
| --- | --- | --- | --- | --- | --- | --- | --- | --- | --- | --- | --- | --- | --- | --- | --- | --- |
| S1 | -0.04 | 0.07 | -0.15 | -0.08 | -0.05 | 0.03 | 0.01 | -0.03 | 0.01 | 0.02 | 0.02 | 0.05 | -0.08 | 0.01 | -0.10 | 0.00 |
| S2 | -0.05 | -0.07 | -0.09 | -0.07 | -0.06 | 0.09 | -0.02 | -0.01 | -0.03 | 0.02 | 0.04 | -0.09 | -0.11 | -0.05 | -0.01 | -0.06 |
| S3 | -0.15 | -0.10 | -0.14 | 0.01 | **-*0.25*** | 0.10 | -0.15 | -0.09 | -0.09 | 0.10 | -0.07 | -0.13 | -0.17 | -0.15 | -0.01 | -0.11 |
| S4 | -0.07 | -0.11 | -0.09 | -0.03 | -0.04 | 0.04 | 0.01 | -0.03 | -0.03 | 0.01 | -0.03 | 0.01 | -0.06 | -0.04 | -0.10 | -0.05 |
| S5 | -0.04 | -0.10 | 0.11 | -0.05 | -0.08 | 0.03 | -0.02 | -0.03 | -0.04 | 0.05 | -0.05 | -0.08 | 0.00 | -0.06 | -0.16 | -0.06 |
| S6 | -0.03 | 0.06 | -0.11 | 0.01 | -0.01 | -0.04 | 0.05 | -0.07 | -0.07 | -0.03 | -0.04 | 0.02 | -0.14 | -0.07 | 0.01 | -0.08 |
| S7 | -0.03 | -0.08 | 0.10 | 0.09 | -0.08 | -0.11 | -0.08 | 0.01 | 0.02 | 0.13 | -0.01 | -0.11 | 0.04 | -0.04 | 0.05 | 0.02 |
| S8 | 0.03 | -0.03 | -0.01 | 0.00 | 0.04 | 0.09 | 0.11 | 0.00 | -0.01 | -0.04 | -0.01 | 0.11 | -0.10 | 0.00 | -0.06 | -0.05 |
| S9 | -0.05 | -0.11 | -0.04 | -0.09 | 0.00 | 0.02 | -0.02 | 0.02 | 0.02 | 0.00 | 0.07 | 0.01 | -0.09 | 0.02 | -0.03 | 0.00 |
|  |  |  |  |  |  |  |  |  |  |  |  |  |  |  |  |  |
|  | **S-F17** | **S-F18** | **S-F19** | **S-F20** | **S-F21** | **S-F22** | **S-F23** | **S-F24** | **S-F25** | **S-F26** | **S-F27** | **S-F28** | **S-F29** | **S-F30** | **S-F31** | **S-F32** |
| S1 | -0.07 | -0.08 | 0.02 | 0.04 | 0.18 | 0.14 | 0.05 | **-0.22** | 0.06 | 0.13 | 0.07 | -0.07 | -0.03 | -0.06 | 0.06 | 0.13 |
| S2 | -0.07 | 0.07 | 0.04 | -0.08 | -0.08 | -0.07 | -0.11 | 0.17 | -0.11 | 0.18 | 0.16 | -0.01 | 0.05 | 0.10 | -0.11 | 0.10 |
| S3 | **-0.23** | 0.08 | 0.11 | -0.19 | 0.09 | 0.06 | -0.08 | 0.10 | 0.01 | 0.16 | -0.06 | **0.22** | 0.04 | 0.05 | 0.01 | **0.20** |
| S4 | -0.01 | -0.11 | 0.06 | -0.07 | 0.03 | 0.05 | -0.12 | -0.08 | -0.01 | 0.05 | 0.04 | -0.04 | 0.07 | 0.07 | -0.01 | 0.08 |
| S5 | -0.06 | 0.02 | 0.14 | -0.11 | 0.10 | 0.16 | -0.10 | -0.05 | 0.04 | 0.00 | 0.10 | 0.09 | 0.15 | 0.16 | 0.04 | 0.02 |
| S6 | 0.00 | -0.01 | -0.02 | -0.08 | 0.18 | 0.15 | 0.04 | -0.03 | 0.05 | 0.05 | 0.03 | 0.01 | -0.05 | -0.10 | 0.05 | 0.08 |
| S7 | -0.06 | -0.07 | 0.01 | -0.07 | -0.03 | -0.02 | -0.03 | 0.03 | 0.05 | 0.12 | 0.20 | 0.04 | 0.07 | 0.14 | 0.05 | 0.05 |
| S8 | 0.09 | -0.11 | 0.00 | -0.12 | 0.06 | 0.05 | 0.03 | 0.04 | 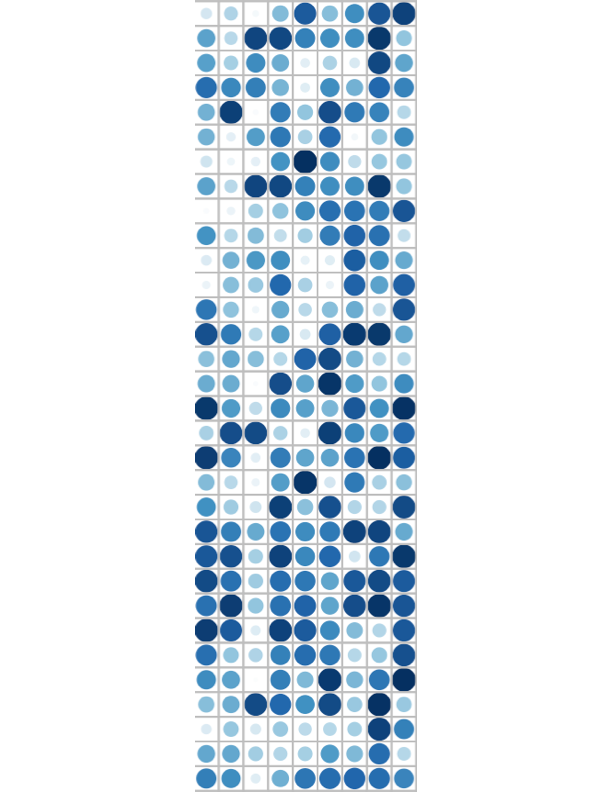-0.01 | 0.09 | -0.09 | -0.04 | -0.03 | 0.01 | -0.01 | 0.02 |
| S9 | 0.05 | -0.11 | 0.07 | -0.02 | 0.02 | 0.07 | -0.12 | -0.02 | 0.09 | -0.09 | 0.05 | 0.11 | 0.04 | 0.07 | 0.09 | 0.01 |
| **BOLD** font indicating \|r\|>.20 and *p*<0.05 (**S-F17–S3**, **S-F24–S1**, **S-F28–S3**, and **S-F32–S3**)  **BOLD** and *Italic* font indicating \|r\|>.20 and FDR p-adjusted<0.05 (***S-F5–S3***)  The background ellipse illustrated the p-values (darker closer to 1 and lighter closer to 0). | | | | | | | | | | | | | | | | |

Table S7 | Correlation of Post>Pre in EMA and Sleep features thresholding at |r|>0.20.

# Reliable Change Index in Wearable

This section reports the reliable change index (RCI) in wearable features, reporting the number of participants with increased, decreased, or no reliable change in activity, readiness and sleep.

1. Activity

| **Feature Code** | **Increased count** | **Decreased Count** | **No Reliable Change** | **Total Count** |
| --- | --- | --- | --- | --- |
| A-F1 | 2 | 0 | 97 | 99 |
| A-F2 | 1 | 3 | 94 | 98 |
| A-F3 | 8 | 10 | 80 | 98 |
| A-F4 | 3 | 3 | 92 | 98 |
| A-F5 | 2 | 4 | 92 | 98 |
| A-F6 | 3 | 1 | 94 | 98 |
| A-F7 | 5 | 5 | 88 | 98 |
| A-F8 | 2 | 1 | 96 | 99 |
| A-F9 | 0 | 0 | 99 | 99 |
| A-F10 | 2 | 1 | 96 | 99 |
| A-F11 | 7 | 7 | 85 | 99 |
| A-F12 | 4 | 0 | 95 | 99 |
| A-F13 | 0 | 3 | 96 | 99 |
| A-F14 | 5 | 4 | 90 | 99 |
| A-F15 | 3 | 1 | 95 | 99 |
| A-F16 | 2 | 1 | 96 | 99 |
| A-F17 | 1 | 1 | 97 | 99 |
| A-F18 | 8 | 4 | 87 | 99 |
| A-F19 | 2 | 1 | 96 | 99 |
| A-F20 | 2 | 5 | 92 | 99 |
| A-F21 | 1 | 0 | 98 | 99 |
| A-F22 | 0 | 1 | 98 | 99 |
| A-F23 | 7 | 4 | 88 | 99 |
| A-F24 | 3 | 2 | 94 | 99 |
| A-F25 | 8 | 2 | 89 | 99 |
| A-F26 | 8 | 2 | 89 | 99 |
| A-F27 | 2 | 2 | 95 | 99 |
| A-F28 | 2 | 2 | 95 | 99 |
| A-F29 | 4 | 0 | 95 | 99 |

Table S8 | RCI for activity features with increased, decreased, no change, and total counts.

1. Readiness

| **Feature Code** | **Increased count** | **Decreased Count** | **No Reliable Change** | **Total Count** |
| --- | --- | --- | --- | --- |
| R-F1 | 11 | 7 | 81 | 99 |
| R-F2 | 3 | 4 | 92 | 99 |
| R-F3 | 16 | 13 | 70 | 99 |
| R-F4 | 2 | 2 | 94 | 98 |
| R-F5 | 9 | 8 | 82 | 99 |
| R-F6 | 10 | 11 | 78 | 99 |
| R-F7 | 14 | 14 | 71 | 99 |
| R-F8 | 5 | 3 | 91 | 99 |
| R-F9 | 16 | 13 | 70 | 99 |

Table S9 | RCI for readiness features with increased, decreased, no change, and total counts.

1. Sleep

| **Feature Code** | **Increased count** | **Decreased Count** | **No Reliable Change** | **Total Count** |
| --- | --- | --- | --- | --- |
| S-F1 | x | 2 | 92 | 99 |
| S-F2 | 8 | 3 | 88 | 99 |
| S-F3 | 4 | 4 | 91 | 99 |
| S-F4 | 0 | 1 | 98 | 99 |
| S-F5 | 9 | 2 | 88 | 99 |
| S-F6 | 9 | 3 | 87 | 99 |
| S-F7 | 7 | 5 | 87 | 99 |
| S-F8 | 9 | 7 | 83 | 99 |
| S-F9 | 14 | 12 | 73 | 99 |
| S-F10 | 6 | 5 | 88 | 99 |
| S-F11 | 13 | 2 | 84 | 99 |
| S-F12 | 6 | 5 | 88 | 99 |
| S-F13 | 1 | 6 | 92 | 99 |
| S-F14 | 9 | 10 | 80 | 99 |
| S-F15 | 6 | 4 | 89 | 99 |
| S-F16 | 15 | 12 | 72 | 99 |
| S-F17 | 8 | 3 | 88 | 99 |
| S-F18 | 11 | 1 | 87 | 99 |
| S-F19 | 4 | 4 | 91 | 99 |
| S-F20 | 4 | 6 | 89 | 99 |
| S-F21 | 0 | 0 | 99 | 99 |
| S-F22 | 0 | 0 | 99 | 99 |
| S-F23 | 9 | 3 | 87 | 99 |
| S-F24 | 1 | 0 | 98 | 99 |
| S-F25 | 16 | 19 | 64 | 99 |
| S-F26 | 1 | 1 | 97 | 99 |
| S-F27 | 0 | 0 | 99 | 99 |
| S-F28 | 1 | 0 | 98 | 99 |
| S-F29 | 4 | 10 | 85 | 99 |
| S-F30 | 5 | 12 | 82 | 99 |
| S-F31 | 16 | 19 | 64 | 99 |
| S-F32 | 3 | 5 | 91 | 99 |

Table S10 | RCI for sleep features with increased, decreased, no change, and total counts.

# Missing Completely at Random

The missing data have been studied using Little’s missing completely at random (MCAR) test to determine whether the missingness occurred randomly. Taking the two-week (14 days) baseline measurement before the VR session and the 12-week (84 days) program period after the VR session. MCAR has been calculated through the course of the 14-week period for the nine EMA scales and 3 wearable measurements. The Oura MCAR studies have been divided into 4 time blocks due to the large number of variables (98 days per participant). The time blocks are 14 days for pre-VR and three 28-day post-VR blocks.

|  | $\boldsymbol{\chi}^{\mathbf{2}}$ | **DF** | ***P*** | **Missing Pattern** |
| --- | --- | --- | --- | --- |
| **Wearable** | | | | |
| Activities | 1076.34 | 1076 | .49 | N/A |
| Readiness | 3913.98 | 3628 | **<.001** | N/A |
| Sleep | 3913.98 | 3628 | **<.001** | N/A |
| **Brief EMA** | | | | |
| GAD-2 | 1564 | 1414 | **.003** | 58 |
| PHQ-2 | 1312 | 1273 | .22 | 53 |
| MIOS-4 | 1306 | 1302 | .47 | 54 |
| SUDS | 1445 | 1428 | .37 | 59 |
| UCLA-3 | 1450 | 1504 | .84 | 62 |
| **Extended EMA** | | | | |
| GAD-7 | 301 | 328 | .86 | 31 |
| PHQ-9 | 331 | 328 | .44 | 31 |
| Brief MIOS | 270 | 333 | .995 | 31 |
| SUDS | 399 | 357 | .06 | 33 |

Table S11 | MCAR to determine the random nature of the missing data.
